# A benzylidene-amine scaffold as a colourimetric sensor for picric acid: computational studies and real-time applications using matchstick head powder

**DOI:** 10.1186/s13065-025-01670-4

**Published:** 2025-11-21

**Authors:** Viswanathan Hemalatha, Sundaramoorthy Sarveswari, Vijayaparthasarathi Vijayakumar

**Affiliations:** Department of Chemistry, School of Advanced Sciences, Vellore Institute of Technology, Vellore, 632014 Tamil Nadu India

**Keywords:** Benzylidene-amine appended probes, ICT, DFT, Paper strips, Matchstick head powder

## Abstract

A series of simple and cost-effective benzylidene-amine appended probes (E)-4-((4-(allyloxy) benzylidene)amino)-N-phenyl aniline (**L-1**), (E)-N-phenyl-4-((2,3,4-trimethoxybenzylidene) amino)aniline (**L-2**), and (E)-4-((2-nitrobenzylidene)amino)-N-phenyl aniline (**L-3**) have been developed for the rapid detection of picric acid (PA) and explored for the same. Notably, **L-3** showed no changes in its UV spectrum or colour upon interaction with PA. In contrast, when PA was added to **L-1** and **L-2**, there were significant changes in absorption, resulting in distinct colour changes. Mole fraction analysis indicated a 1:1 ratio between the probes and PA. The detection limits for PA were impressively low, measuring 4.37 × 10^− 7^ M for **L-1** and 4.56 × 10^− 6^ M for **L-2**. This demonstrates the effectiveness of these probes in detecting PA, even at very low concentrations. Importantly, this work marks the first application of benzylidene-amine probes for the detection of PA. The sensing studies were supported by theoretical analyses and calculations, which confirmed the selective detection of PA using matchstick head powder (MSPS) and a test kit based on paper strips. Additionally, smartphone-assisted detection using an RGB tool was successfully demonstrated.

**Graphical abstract:**

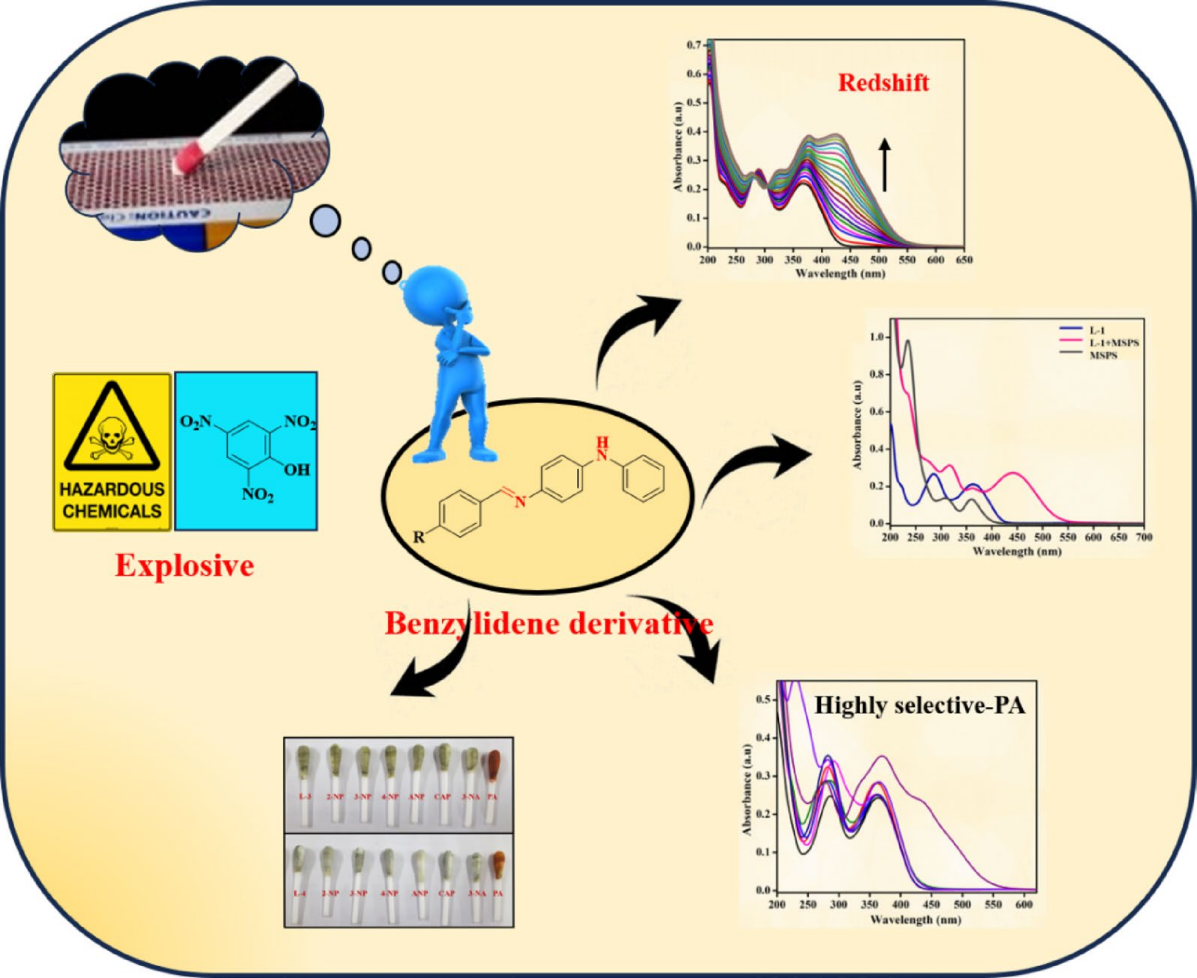

**Supplementary Information:**

The online version contains supplementary material available at 10.1186/s13065-025-01670-4.

## Introduction

Picric acid (PA) is a well-known for its strong explosive properties [1–3] and is widely utilized in various industries, including explosives, plasticizers, matchbox production, and pharmaceuticals [4–6]. However, it is also a significant groundwater contaminant due to its high solubility in water. Prolonged exposure to PA can result in numerous health issues, such as skin and eye inflammation, liver impairment, and long-term conditions like nausea, liver damage, anaemia, dizziness, and cyanosis [7]. The allowable limit of PA in water is set at 2.2 µM, with concentrations exceeding this limit posing a risk of environmental pollution that can affect soil, rivers, water bodies, and agricultural land, ultimately harming aquatic life [8, 9]. Traditional methods for detecting PA, such as chromatography coupled with atomic absorption spectroscopy, energy-dispersive X-ray diffraction, graphite furnace atomic absorption spectrometry, mass spectrometry, hybrid generation atomic absorption spectroscopy, surface-enhanced Raman spectroscopy, and nuclear quadrupole resonance spectroscopy, tend to be complex, time-consuming, and require expensive equipment [10, 11]. In contrast, the emergence of colorimetric detection technology offers a more cost-effective and immediate approach with high specificity [12, 13]. In response to the demand for rapid and sensitive organic detectors, there has been increasing interest in the design and synthesis of colorimetric chemosensors. These chemosensors typically incorporate core moieties such as Schiff bases, pyridine, pyrazole, pyrene, anthracene, rhodamine, quinoline, naphthalene, thiourea, urea, and coumarin, demonstrating promising selectivity, sensitivity, and performance [14–20].

Advancements in chemical sensors designed for the selective colorimetric identification of explosive nitroaromatics, particularly PA, present significant challenges but are essential due to the serious environmental and health implications. Sensors capable of detecting PA in diverse environments including explosive zones, arms storage facilities, wastewater treatment areas, and agricultural irrigation fields, are crucial. To address these challenges, we have designed and synthesized new benzylidene-amine-appended probes. Among these, probes **L-1** and **L-2** demonstrated a colorimetric response to PA with minimal interference from other nitroaromatics, highlighting their potential for selective detection. The detection process involves interactions between the hydroxyl group of PA and the secondary amine group(s) of the probes. Additionally, these probes demonstrated high selectivity toward PA at low concentrations in a CH_3_CN medium, while **L-3** showed no response to PA. Furthermore, we introduced a strategic approach using powdered matchstick heads for real sample analysis without the need for spiking detection, offering a new dimension to the detection process.

## Experimental section

### Materials and methods

The materials used in this research were obtained from suppliers and used without further purification. HPLC-grade acetonitrile was employed for analytical investigations. Absorption measurements were conducted using a Jasco V-670 spectrometer in a CH_3_CN medium. TMS was utilized as an internal standard for the NMR spectra, which were recorded using a Bruker Avance 400 MHz instrument. The IR spectrum was recorded with a Shimadzu FT-IR Affinity-1 Fourier transform infrared spectrometer. ESI-MS was performed using a Water Xevo G2 XS-ToF instrument, and GC-MS analyses were conducted with Perkin Elmer Clarus 680 and 600 instruments. Additionally, DFT calculations were carried out using Gaussian 6.1 with the 16 W software packages.

### Synthesis and characterization of probes L-1 – L-3

**Scheme 1:**
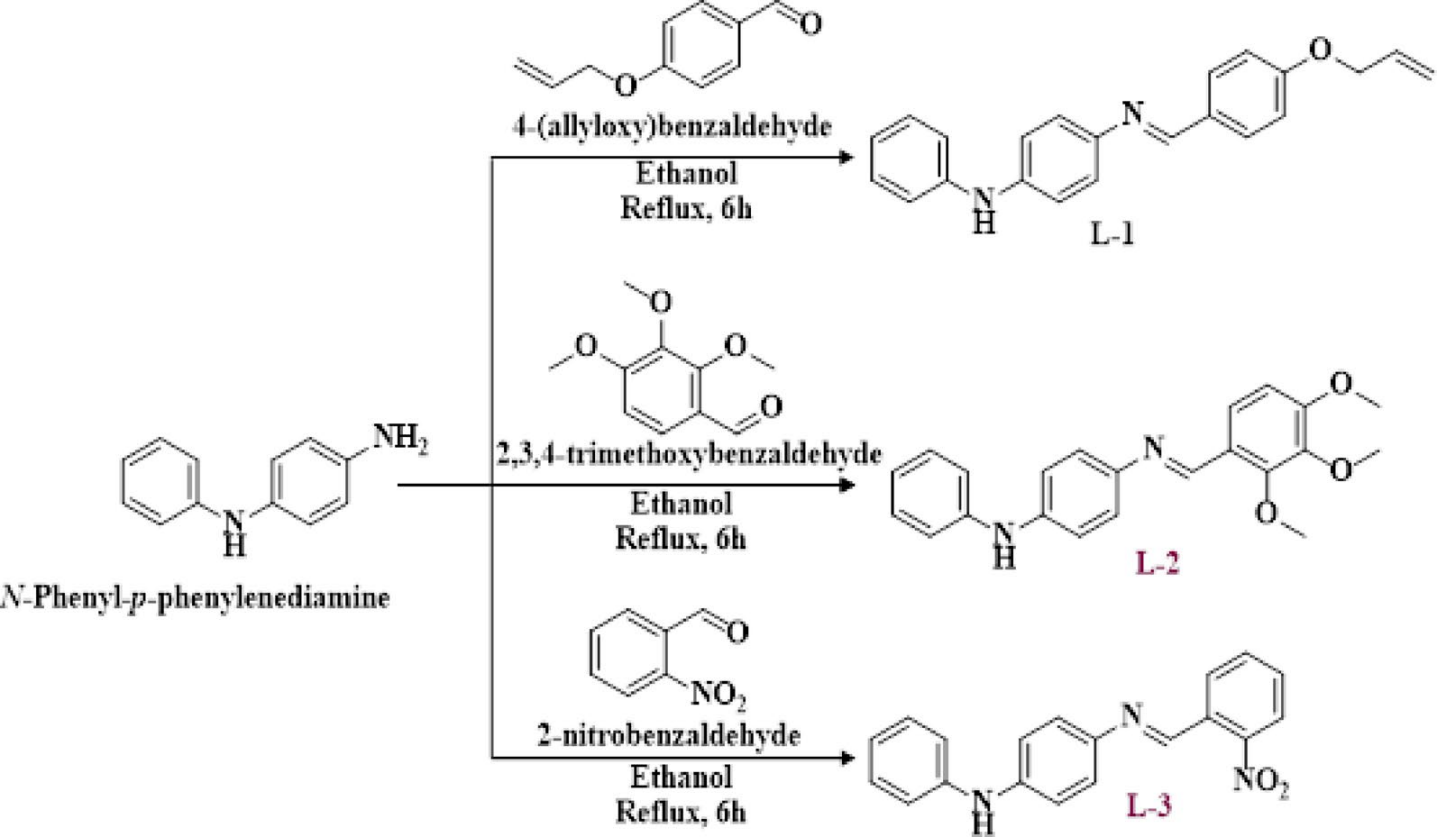
Synthetic pathways for **L-1**,** L-2** and **L-3**

The required probes, **L-1**,** L-2** and **L3**, were synthesized by reacting N-phenyl-*p*-phenylenediamine with the appropriate aryl aldehydes, as shown in Scheme 1. The probe (E)-4-((4-(allyloxy)benzylidene)amino)-N-phenyl aniline (**L-1**) is an amine-appended benzylidene, synthesized by reacting 0.5 g of the 4-allyloxy aldehyde with 0.56 g of N-phenyl-*p*-phenylenediamine in ethanol (25 mL) under reflux for 6 h (see Scheme 1). The progress of the reaction was monitored using thin-layer chromatography (TLC). Once the reaction was complete, the obtained crude mass was thoroughly washed with hexane and filtered. The purity of the solid product was checked using TLC, and its melting point was recorded using an open capillary tube. Similarly, probes **L-2** and **L-3** were synthesized by reacting 2,3,4-trimethoxybenzaldehyde and 2-nitrobenzaldehyde, respectively, with N-phenyl-*p*-phenylenediamine in a 1:1 molar ratio (as illustrated in Scheme 1). The structures of the synthesized probes were confirmed using IR, ^1^H NMR, ^13^C NMR, DEPT-135, H, H-COSY, HSQC, and HR-MS spectral data.

The compound **L-1** has been considered as a representative example and its unambiguous chemical shift assignment using IR, ^1^H NMR, ^13^C NMR, DEPT-135, H, H-COSY, HSQC, and HR-MS spectral data has been described as follows. ^1^H and ^13^C NMR spectra (Fig. S1-S3) of compound **L-1** was thoroughly examined, and the findings are presented in the spectral characterization section. Proton bearing and non-proton bearing carbons are identified, and segregated into primary, secondary and tertiary carbons using DEPT-135 (Fig. S4). A careful analysis of the H, H-COSY spectrum (Fig. S5-S6) revealed the following: The signal at δ 8.42 ppm, integrating for one proton and appearing as a singlet, is attributed to the proton at the imine carbon. The HSQC analysis indicated that the corresponding carbon appears at δ 157.5 ppm. The doublet at δ 7.83 ppm (*J* = 8.80 Hz) couples with another doublet at δ 6.99 ppm (*J* = 8.80 Hz), each integrating for two protons and assigned to the *meta* and *ortho* protons of the allyloxy-substituted phenyl group, with the corresponding carbons located at δ 130.2 and δ 114.9 ppm in HSQC (Fig. S7-S8). *Meta* and *ortho* protons of aryl ring B appeared at δ 7.20 (d, *J* = 8.80 Hz, 2 H) and δ 7.09 (d, *J* = 8.80 Hz, 2 H), with their corresponding carbons found at δ 122.2 and δ 118.7 ppm, respectively. Signals for the unsubstituted phenyl group were found at δ 7.26 (t, *J* = 7.20 Hz, 2 H), δ 7.07 (d, *J* = 8.00 Hz, 2 H), and δ 6.92 (t, *J* = 7.20 Hz, 1H). The signal at δ 7.26 was found to couple with the signals at δ 7.07 and δ 6.92, identifying them as meta protons. The doublet at δ 7.07 ppm corresponds to the *ortho* protons, while the triplet at δ 6.92, integrating for one proton, represents the para proton. Their respective *meta*,* ortho*, and *para* carbons were located at δ 129.4, δ 117.5, and δ 120.9 ppm in HSQC. Additionally, the remaining five non-proton bearing carbon atoms at the ipso and para positions of all three probes were observed at δ 129.7, δ 141.2, δ 143.3, δ 145.6, and δ 161 ppm. The observation of stretching frequencies for –NH, C = N, and C = C groups at 3416 cm⁻¹, 1602 cm⁻¹, and 1511 cm⁻¹, respectively, along with the absence of primary amine and aldehyde frequencies in the IR spectrum (Fig. S9) of compound L-1, confirms the successful formation of the target molecule. The observed m/z value at 327.1371 in the HR-MS spectrum (Fig. S10) also supports this conclusion. The other two compounds, L2 and L3, were also characterized, and their spectral characterization data can be found in the “Spectral Characterisation” section.

####  Spectral characterization of L-1

Grey solid, yield 96%, m.p.: 85–87 °C; ^1^H NMR (400 MHz, CDCl_3_): *δ* ppm 8.42 (s, 1H), 7.83 (d, *J* = 8.80 Hz, 2 H, *meta* protons of allyloxy substituted phenyl), 7.26 (t, *J* = 7.20 Hz, 2 H *meta* protons of unsubstituted phenyl), 7.20 (d, *J* = 8.80 Hz, 2 H, *meta* protons of the aryl ring B), 7.09 (d, *J* = 8.80 Hz, 2 H, *ortho* protons of the aryl ring B), 7.07 (d, *J* = 8.00 Hz, 2 H, *ortho* protons unsubstituted phenyl), 6.99 (d, *J* = 8.80 Hz, 2 H, *ortho* protons of allyloxy substituted phenyl), 6.92 (t, *J* = 7.20 Hz, 1H *para* protons unsubstituted phenyl), 6.07 (ddd, 17.26, 10.52, 5.2 Hz, CH proton of alkene in allyl), 5.75 (s, 1H, NH proton), 5.43 (dd, *J* = 17.26, 1.60 Hz, 1H, *trans* proton of terminal CH_2_), 5.32 (dd, *J* = 10.40, 1.20 Hz, 1H, *cis* proton of terminal CH_2_), 4.60 (d, *J* = 5.20 Hz, 2 H, CH_2_ of the allyl). ^13^C NMR (100 MHz, CDCl_3_): *δ* ppm 161.0, 157.5, 145.6, 143.3, 141.2, 132.8, 130.2, 129.7, 129.4, 122.2, 120.9, 118.7, 118.0, 117.5, 114.9, 68.8. DEPT-135˚ NMR: *δ* ppm 157.5 (imine carbon), 132.8 (-CH carbon of alkene of allyl), 130.2 (*meta* carbons of allyloxy substituted phenyl), 129.4 (*meta* carbon of unsubstituted phenyl), 122.2 (*meta* carbon of aryl ring), 120.9 (*para* carbon of unsubstituted phenyl), 118.7 (*meta* carbon of aryl ring), 118.0 (-CH_2_ carbon of terminal alkene of allyl), 117.5 (*ortho* carbon of unsubstituted phenyl), 114.9 (*ortho* carbons of allyloxy substituted phenyl), 68.8 (-CH_2_ carbon of allyl), IR ν/cm^− 1^ = 3416 (NH), 1602 (C = N), 1511 (C = C). HR-MS [M]^+^ C_22_H_20_N_2_O; expected mass: 328.4150, found mass [M-1]^+^: 327.1371.

#### Spectral characterization of L-2

Green solid, yield 98%, m.p.: 102–104 °C; ^1^H NMR (400 MHz, CDCl_3_): *δ* ppm 8.77 (s, 1H, proton at -N = CH), 7.88 (d, *J* = 9.0 Hz, 1H, *ortho* proton of trimethoxy phenyl), 7.28 (t, *J* = 8.0 Hz, 2 H, *meta* protons of unsubtituted phenyl), 7.22 (d, *J* = 8.4 Hz, 2 H, meta protons of aryl at ring B), 7.11 (d, *J* = 8.4 Hz, 2 H, *ortho* protons of aryl at ring B), 7.08 (d, *J* = 8.0 Hz, 2 H, *ortho* protons of unsubtituted phenyl), 6.93 (t, *J* = 7.2 Hz, 1H, *para* proton at unsubtituted phenyl), 6.78 (d, *J* = 9.0 Hz, 1H, *meta* proton of trimethoxy phenyl), 5.76 (s, 1H, -NH), 3.97 (s, 3 H, -OCH_3_), 3.93 (s, 3 H, -OCH_3_), 3.90 (s, 3 H, -OCH_3_). ^13^C NMR (100 MHz, CDCl_3_): *δ* ppm 156.2, 154.4, 153.7, 146.0, 143.3, 141.8, 141.2, 129.4, 123.2, 122.4, 122.3, 120.9, 118.7, 117.5, 107.9, 62.0, 60.9, 56.1 (The signals at 156.2, 154.4, 146.0, 143.3, 141.8, 141.2, 123.2 are non proton bearing carbons of *ipso* of phenyl, *ipso* and *para* of aryl at B ring and C1, C2, C3, C4 of trimethoxy phenyl ring since these are not appeared in DEPT-135 spectrum). DEPT-135˚ NMR: *δ* ppm 153.7 (carbon at -N = CH), 129.4 (C-6 of trimethoxy phenyl), 122.4 (*meta* carbons of unsubstitted pheryl), 122.3 (*meta* carbons of aryl at ring B), 120.9 (*para* carbons of unsubstitted pheryl), 118.7 (*ortho* carbons of aryl at ring B), 117.5 (*ortho* carbons of unsubtituted phenyl, 107.9 (C-5 of trimethoxy phenyl), 62.0 (s, 3 H, -OCH_3_), 60.9 (s, 3 H, -OCH_3_), 56.1(s, 3 H, -OCH_3_) IR ν/cm^− 1^ = 3263 (-NH), 1590 (C = N). HR-MS [M]^+^ C_22_H_22_N_2_O_3_; expected mass: 362.4290, found mass [M + 1]^+^: 363.1710.

#### Spectral characterization of L-3

Red solid, yield 95%, m.p.: 92–94 °C; ^1^H NMR (400 MHz, CDCl_3_): *δ* ppm 8.98 (s, 1H, proton at -N = CH), 8.32 (d, *J* = 7.6 Hz, 1H, proton at C-3 of nitro aryl ring-*ortho* to -NO_2_ group), 8.04 (d, *J* = 8.0 Hz, 1H, proton at C-6 of nitro aryl ring, meta to-NO_2_ group at C-6), 7.70 (t, *J* = 7.2 Hz, 1H, proton at C-4 of nitro aryl ring, meta to-NO_2_ group at C-4), 7.57 (t, *J* = 7.6 Hz, 1H, proton at C-5 of nitro aryl ring), 7.29 (t, *J* = 8.0 Hz, 4 H, *meta* of phenyl ring and two protons of aryl of B ring merged and integrated for four protons), 7.10 (d, *J* = 6.0 Hz, 4 H, *ortho* of phenyl ring and two protons of aryl of B ring merged and integrated for four protons), 6.96 (t, *J* = 7.2 Hz, 1H, para proton of phenyl ring), 5.86 (s, 1H, -NH). ^13^C NMR (100 MHz, CDCl_3_): *δ* ppm 152.4, 149.2, 143.6, 142.8, 142.5, 133.4, 131.4, 130.7, 129.5, 129.4, 124.6, 123.0, 121.6, 118.3, 117.7. DEPT-135˚ NMR: *δ* ppm 152.4 (carbon at -N = CH), 133.4 (C4 of nitro aryl), 130.7 (C5 of nitro aryl), 129.5 (C3 of nitro aryl), 129.4 (*meta* of phenyl), 124.6 (C6 of nitro aryl), 123.0 (meta of aryl at ring B), 121.6, 118.3, 117.7. IR ν/cm^− 1^ = 3383, 1585, 1515, 1490, 1344, 1314. GC-MS [M] ^+^ C_19_H_15_N_3_O_2_: expected mass: 317.3480, found mass: 317.3732.

### UV–Vis absorption studies of probes with nitro aromatics

For the UV-Vis absorption studies, a working probe solution of 1 × 10⁻⁵ M and a nitroaromatic solution of 1 × 10⁻³ M were prepared. Changes in the probe signals were recorded using UV-Vis absorption spectra. Probe **L-3** did not show any response towards PA, which is attributed to the presence of the electron-deficient nitro group at the ortho position. Therefore, the photophysical properties of probes **L-1** and **L-2** were further explored.

### Interference studies of probes with nitro aromatics

**Fig. 1:**
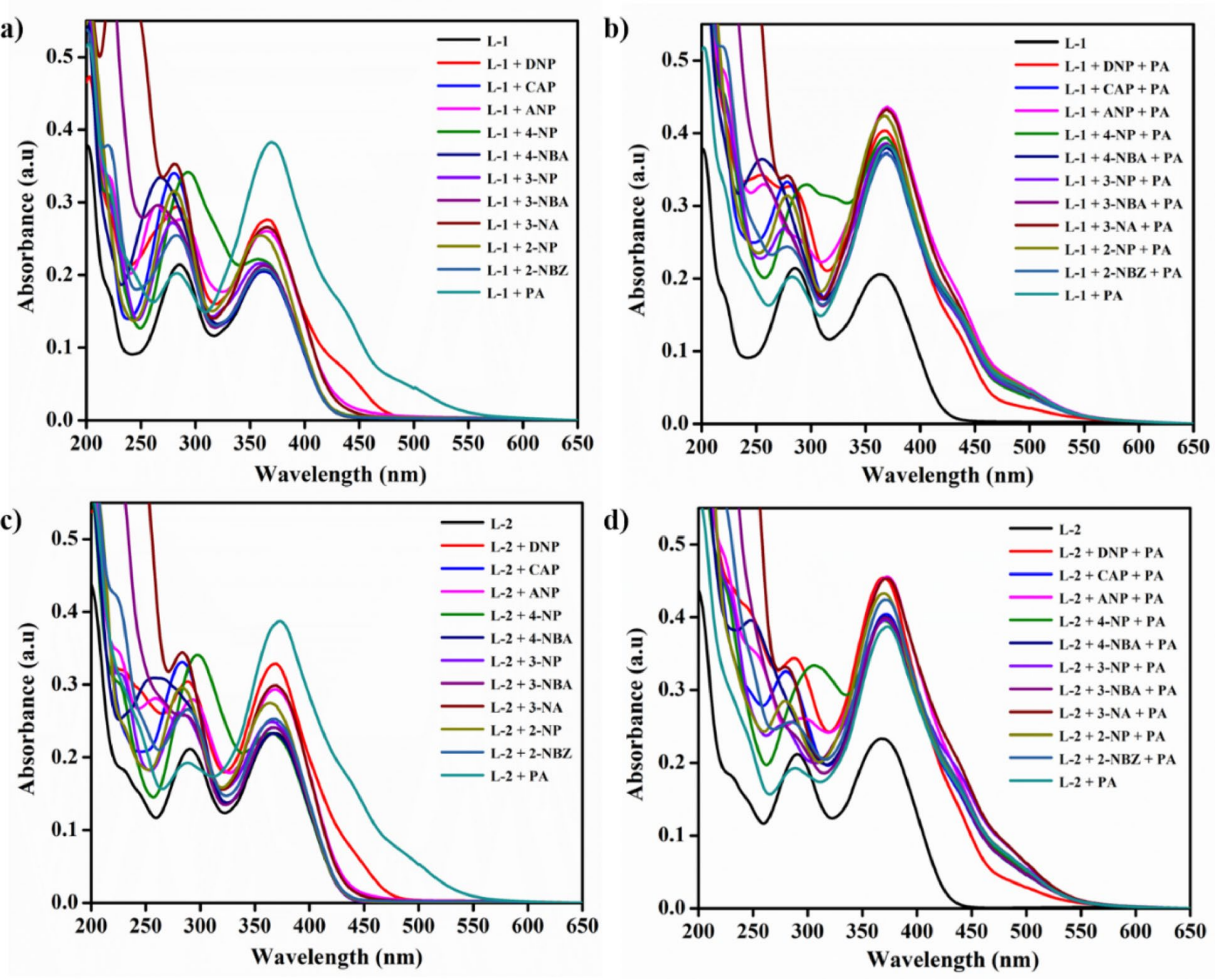
Absorption studies of **a** selectivity and **b** interference of **L-1**, Absorption studies of **c** selectivity and **d** interference of **L-2**

To investigate the interference of these probes with various analytes, 2 mL of a 1 × 10⁻³ M probe aliquot was combined with 20 µL of PA (1 × 10⁻³ M) and 20 µL of nitroaromatic solution (1 × 10⁻³ M) to create mixtures of **L-1** and **L-2** with PA and different nitroaromatics. Changes in the absorption spectrum were subsequently observed, as illustrated in Fig. 1 (b and d).

### Calculation of detection limit (DL) and binding constant (K_a)_ by UV-Vis Titration

The detection limit of the probes for PA was calculated using the formula 3σ/slope [21, 22]. The binding constant was determined through UV-Vis absorption titration, using the Benesi-Hildebrand plot. The association constant (Ka) was derived from the equation: Binding constant (Ka) = I/slope [23, 24]. Here the slope is calculated from the linear relationship between (A-A₀) and [PA], where A₀ represents the absorption of the probe in the absence of PA, and A denotes the absorption measured in the presence of PA.

### Titration studies of probes toward PA

A stock solution was prepared containing **L-1** and **L-2** at a concentration of 1 × 10⁻³ M, along with PA (1 × 10⁻³ M). A 20 µL sample of this stock solution was added to 2 mL of CH_3_CN in a cuvette. The solution was titrated with PA (1 × 10⁻³ M) by incrementally adding 2 µL of PA, up to a total of 38 µL. Changes in the absorption spectrum were recorded over a wavelength range of 250–550 nm.

### Job’s plot

Job’s method was utilized to determine the binding behaviour of probes **L-1** and **L-2** with PA in a CH_3_CN medium. Solutions containing probes (1 × 10⁻⁴ M) and PA (1 × 10⁻⁴ M) were prepared separately. Varying volumes of probe solutions (1.8, 1.6, 1.4, 1.2, 1, 0.8, 0.6, 0.4, and 0.2 mL) were transferred into a 2 mL cuvette, followed by the addition of increasing volumes of PA solution (0.2, 0.4, 0.6, 0.8, 1, 1.2, 1.4, 1.6, and 1.8 mL). The solutions were mixed, and spectra were obtained at ambient temperature to determine the probe-PA association ratio.

### Computational studies

DFT calculations using the Gaussian 6.1 program were employed to optimize the electronic structures of these derivatives and ligands with PA [25–27].

## Results and discussion

The probes **L-1**, **L-2**, and **L-3** were synthesized using a simple one-step procedure, as outlined in Scheme 1. Their synthesis was confirmed through FT-IR, NMR, and LC/HRMS spectral data. The experimental section provides the analytical data for the synthesized compounds, while the spectral evidence can be found in Fig. S1- S30 in the supplementary information.

### Selectivity and sensitivity studies with nitroar

Electron-rich groups or substituents such as primary amines, isopropyl amines, dialkyl amines, and aryl amines that donate electrons and tend to interact with electron-accepting nitroaromatics. To explore this, we investigated the binding behaviour of the synthesised probes towards various nitro compounds such as 4-nitrobenzoic acid (4-NBA), 2,4-dinitrophenol (2,4-DNP), 3-nitrobenzoic acid (3-NBA), 2-nitrobenzaldehyde (2-NBZ), 2-nitrophenol (2-NP), chloramphenicol (CAP), 3-nitrophenol (3-NP), picric acid (PA), 2-amino-4-nitrophenol (ANP), 4-nitrophenol (4-NP), and 3-nitroaniline (3-NA). This investigation was conducted using absorption spectra collected both with and without the addition of 20 µL of the nitro compounds in acetonitrile. Probes **L-1** and **L-2** exhibited a colour change upon the addition of PA, while **L-3** showed no spectral or colour change. This lack of change is likely due to the presence of the nitro group, as shown in Fig.S31. As illustrated in Fig.S31, competitive experiments and interference studies demonstrated that the presence of other nitro aromatics did not hinder the detection of PA. The bar diagram representing the probes is depicted in Fig. S32.

**Fig. 2:**
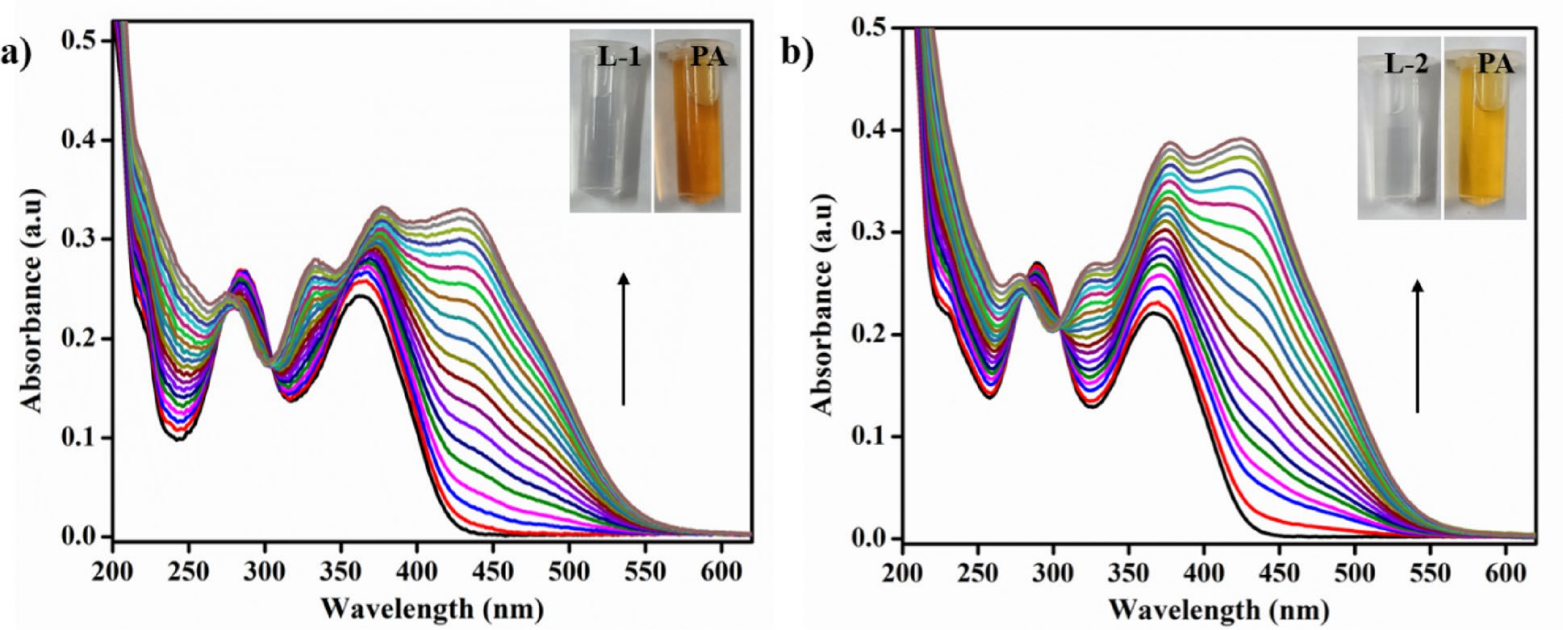
**a** L-1 UV-Vis titration and **b ** L-2 the gradual increment of PA (0–38 µL) in CH_3_CN

In the sensitivity studies of the probes, increments of 2 µL of PA (ranging from 0 to 38 µL) were separately added to the acetonitrile solution of each probe. As shown in Fig. 2a, L-1 exhibited a decrease in absorption intensity at 286 nm and 362 nm, while an increase in absorption intensity was observed at 428 nm. In Fig. 2, L-2 initially displayed absorption maxima at 289 nm and 366 nm. After the addition of PA, both of these absorption maxima decreased, and a new peak emerged with maximum absorption at 424 nm. These changes in the spectral characteristics were attributed to the interaction between PA and the probes.

### Detection limit, binding studies of probes

**Fig. 3:**
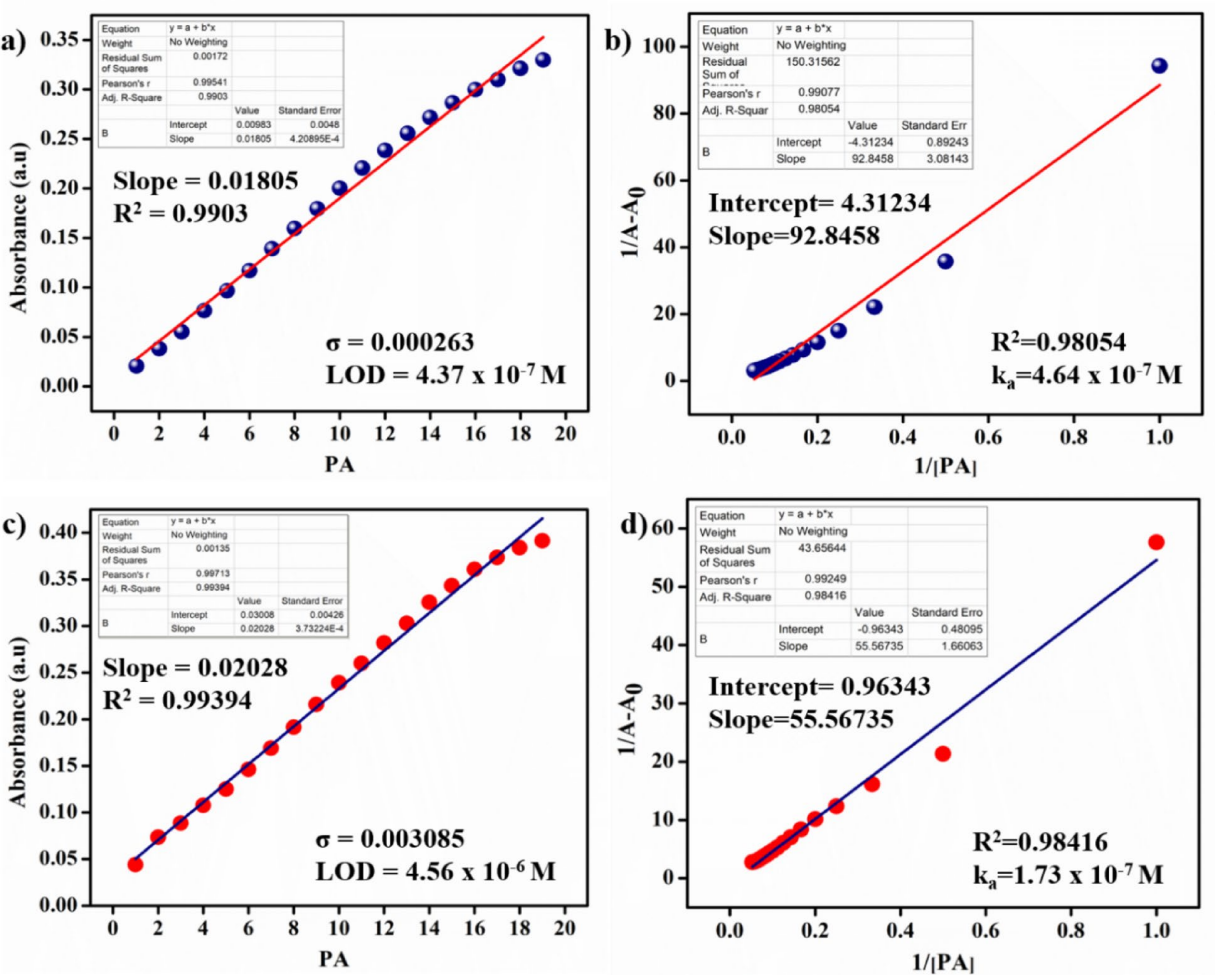
**a** **L-1** The detection limit (LOD) and **c** **L-2**, and Benesi–Hildebrand plots of **b** **L-1 + PA** at 428 nm and **d** **L-2 + PA** at 424 nm

The detection limit values for **L-1** (4.37 × 10^− 7^ M, Fig. 3a) and **L-2** (4.56 × 10^− 6^ M, Fig. 3c) were determined by analysing changes observed in the absorption spectrum. The detection limit (DL) was calculated using the Eq. 3σ/s, where the variable ‘s’ is derived from the plot of the change in absorption against the concentration of PA, and ‘σ’ represents the standard deviation of the blank probe (Fig.S32) recorded in the absence of PA. Furthermore, the association constants for **L-1** (Fig. 3b) and **L-2** (Fig. 3d) were determined using the Benesi-Hildebrand equation, yielding values of 4.64 × 10^− 7^ M and 1.73 × 10^− 7^ M, respectively.

**Fig. 4:**
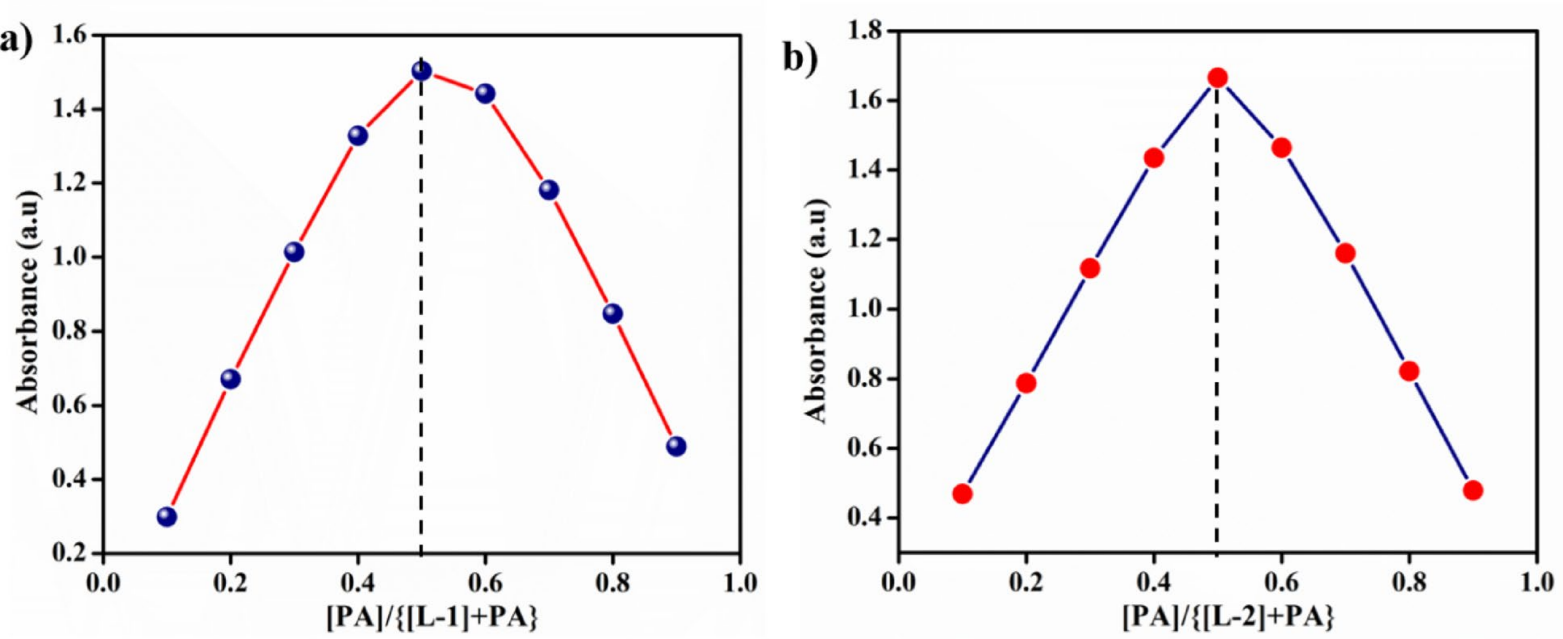
The Job′s plot of **a** **L-1** and **b** **L-2**

The hydroxyl group of PA and the secondary amine of the derivatives may facilitate intermolecular charge transfer (ICT), leading to a significant redshift and a corresponding colour change. Job’s plot determined that the ratio of probes to PA is 1:1, as depicted in Fig. 4. To confirm the formation of the adduct, a ^1^H NMR titration experiment was conducted to provide evidence of the sensing mechanism.

**Fig. 5:**
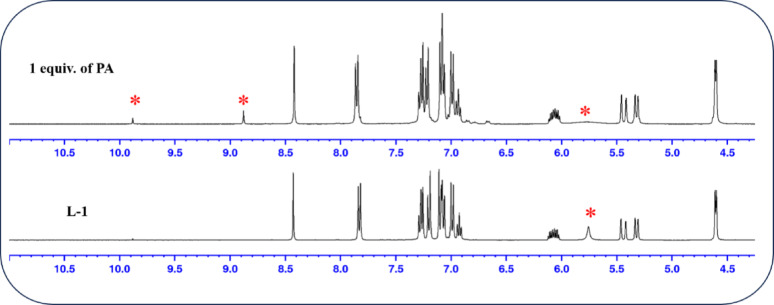
^1^H NMR titration spectrum of **L-1** with PA

**Fig. 6:**
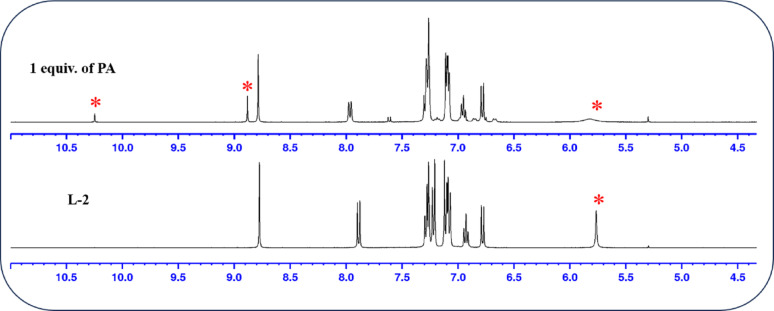
^1^H NMR titration spectrum of **L-2** with PA

To elucidate the interaction sites between the probes and PA, ^1^H NMR titration was performed using PA in CDCl_3_ solvent. This provided strong evidence for the proposed interaction between the probe and PA components. Initially, the ^1^H NMR spectra of the probes were recorded, followed by the addition of PA to the solution (in CDCl_3_). The peak corresponding to the -NH group of the probes appeared at δ 5.75 ppm. Upon adding PA, the signal at δ 5.75 ppm disappeared, indicating that a strong hydrogen bond was formed between the secondary amine group of the probe and the -OH group of PA. The NMR titration spectra are illustrated in Fig. 5. and Fig. 6. Additionally, the -OH proton and aromatic protons of PA appeared at 9.88 and 8.87 ppm for L-1, and at 8.88 and 10.25 ppm for **L-2**, respectively. Most of the aromatic protons of the probes exhibited a downfield shift, which indicates an electrostatic interaction and ICT between PA and the probes. No other changes were observed, confirming the interaction of the hydroxyl group with the secondary amine proton. The electrostatic interaction between the derivatives and PA enhanced the ICT process throughout the molecule, resulting in a significant redshift in the absorption spectrum.

The limit of detection (LOD) of L-1 is lower compared to L-2. This is primarily due to the allyloxy group in L-1, which is small and flexible, allowing enhanced intramolecular charge transfer (ICT) through favorable electrostatic interactions. In contrast, L-2 contains three methoxy groups at positions 2, 3, and 4, which introduce significant steric hindrance which disrupts molecular planarity and weakens ICT efficiency, resulting in a higher LOD than L-1.

**Scheme 2:**
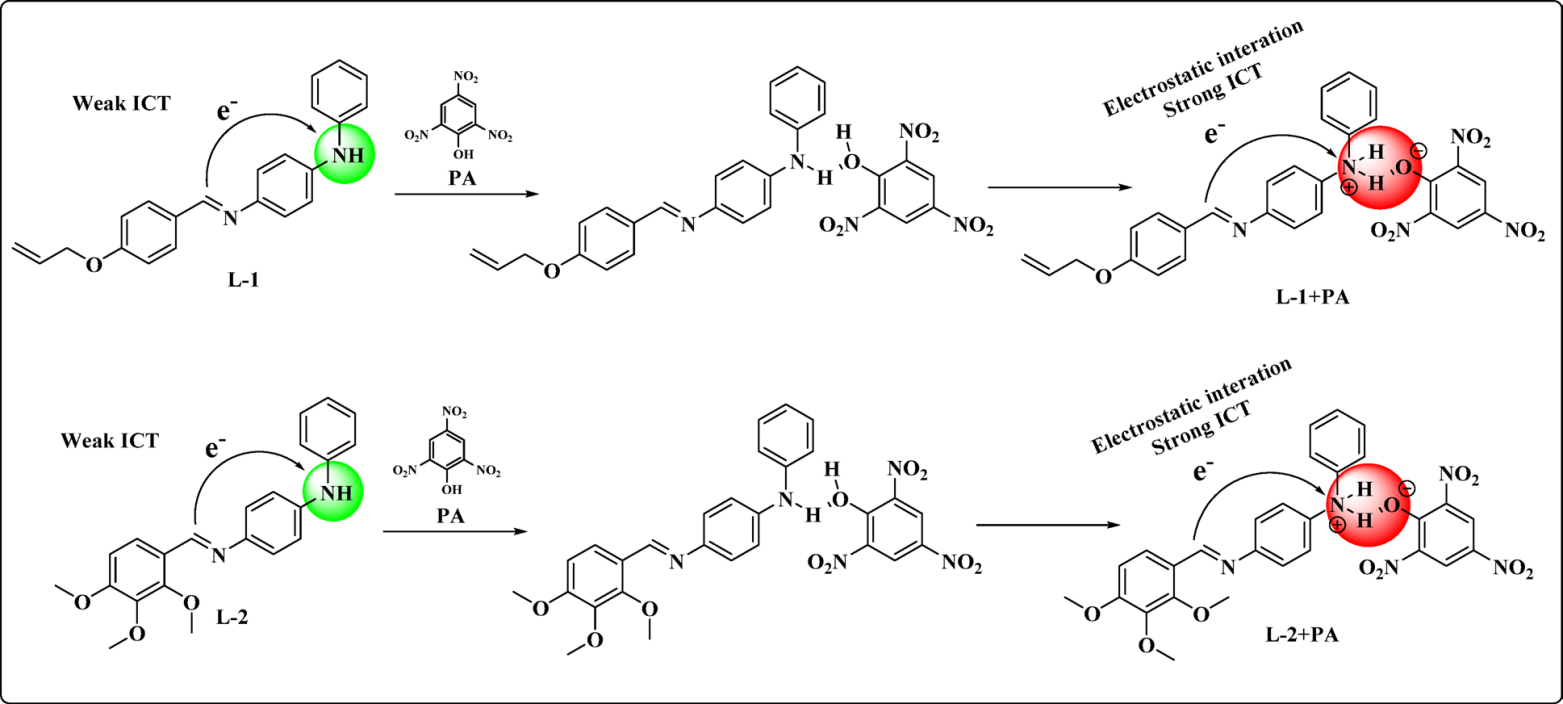
Probable binding mechanism of the **L-1 + PA** and **L-2 + PA**

The probable binding mechanism of the probes with PA is illustrated in Scheme 2, while the possible outcomes of the probes are presented in Table 1.

**Table 1:** Comparative outcome of probes

Properties and outcomes	L-1	L-2
λ_Max_ of the probe	286 nm, 362 nm	289 nm, 366 nm
λ_Max_ of the probe with PA	428 nm	424 nm
LOD	4.37 × 10^− 7^ M	4.56 × 10^− 6^ M
Binding constant	k_a_= 4.64 × 10^− 7^ M	k_a_=1.73 × 10^− 7^ M
Binding ratio (Probe: Analyte)	1:1	1:1

### Time-dependent and pH effect of L-1 and L-2

**Fig. 7:**
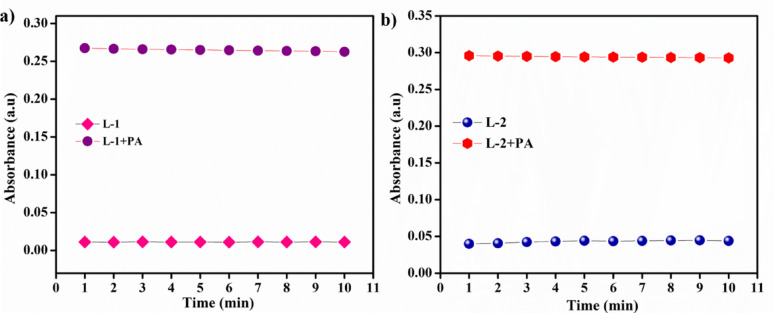
Time-dependent fluorescence spectra of **aL-1** and **b** **L-2**

To investigate the changes in absorbance of **L-1** and **L-2** in detecting PA, we conducted time-dependent absorbance experiments in CH_3_CN. As illustrated in the Fig. 7, both **L-1** and **L-2** exhibited minimal changes in absorbance intensity and maintained stability. Upon the addition of PA, the absorbance intensity increased and reached a plateau after one minute. This finding, shown in Fig. 7, demonstrates that **L-1** and **L-2** can effectively detect PA while maintaining good stability [28–30].

**Fig. 8:**
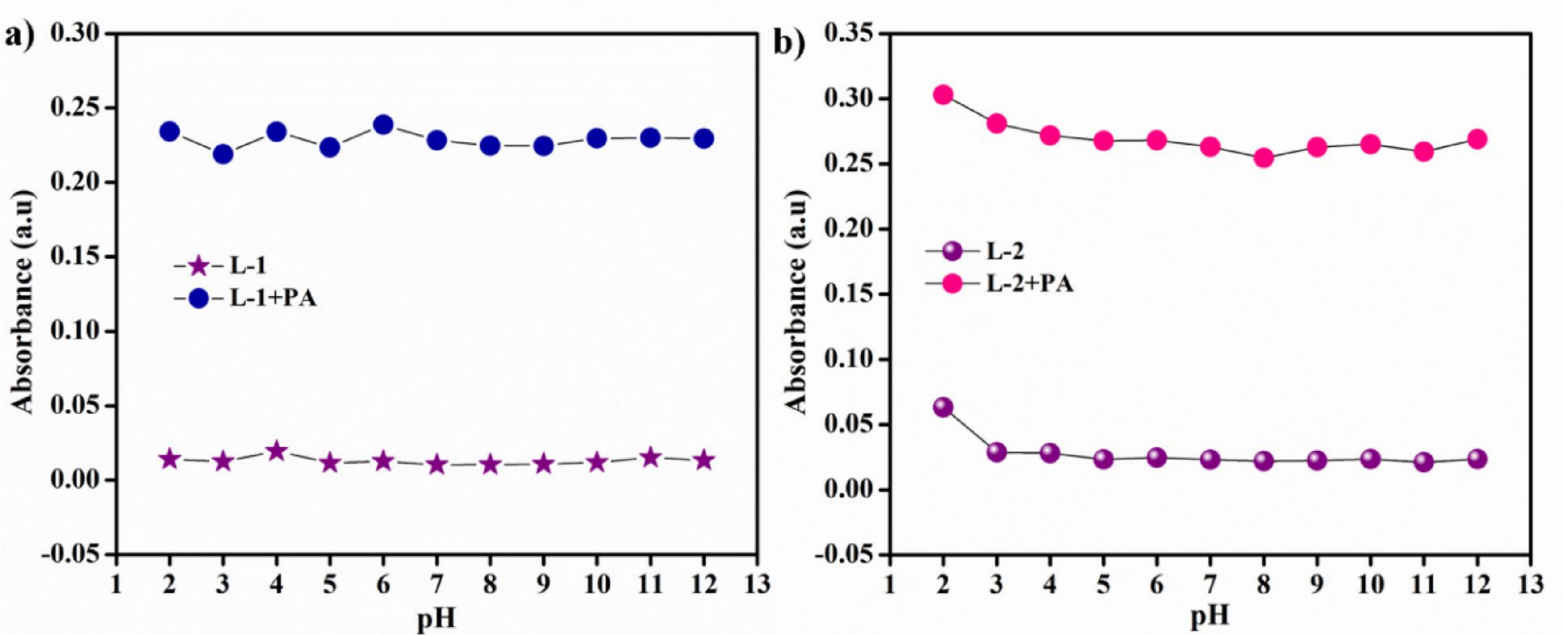
Effect of pH response of **a** L-1 and **b** L-2 in the presence and absence of PA (pH = 2–12)

We also studied the impact of pH on the detection properties of **L-1** and **L-2**, with pH values ranging from 2 to 12, using UV-Vis spectroscopy. Initially, we measured the absorption maxima of the free probe solutions in various pH buffer solutions. We then added 20 µL of PA to the L-1 and L-2 solutions and recorded the corresponding absorption maxima under the same conditions. As shown in Fig. 8. the observed absorption maxima indicate that probes **L-1** and **L-2** can detect PA across the entire pH range of 2 to 12 [31–33].

### Computational study

**Fig. 9:**
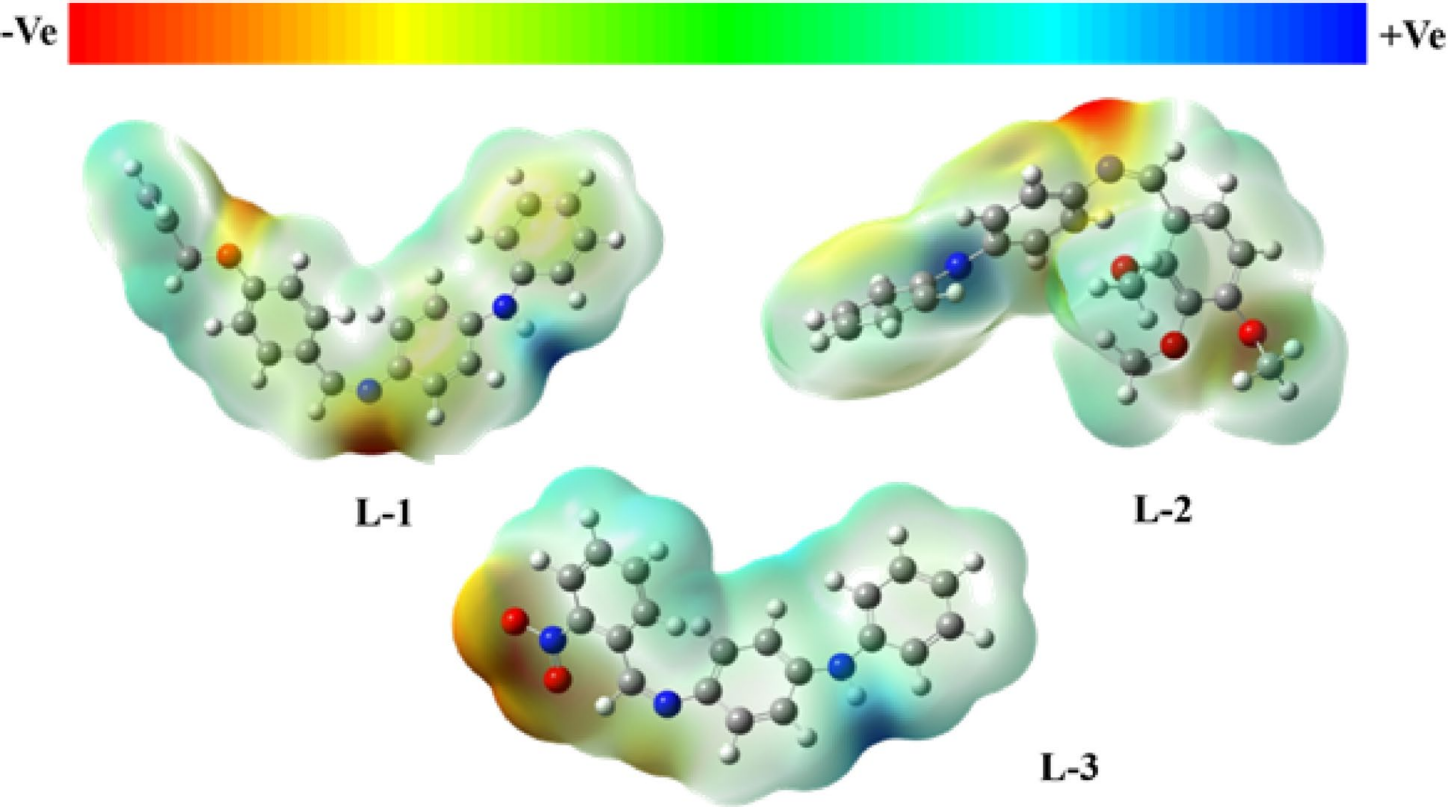
The molecular electrostatic potential surfaces of probes

Quantum chemical methods were used to investigate the electronic states and structural properties of various derivatives. The study employed density functional theory with B3LYP and the 6-311 + G basis set, using the Gaussian G16 software [34]. The geometrical configurations of **L-1**, **L-2**,** L-1** + PA, and **L-2** + PA are summarized in Table S1. Additionally, the electrostatic potential (ESP) mappings of **L-1**, **L-2**, and **L-3**, are shown in Fig. 9, illustrate the charge distributions of these molecules. The ranges of electrostatic potential ranges for the gas phase probes are as follows: **L-1** and **L-2** range from − 5.507e-2 to -5.507e-2 and − 5.824e-2 to 5.824e-2, while **L-3** ranges from − 7.890e-2 to 7.890e-2.

**Fig. 10:**
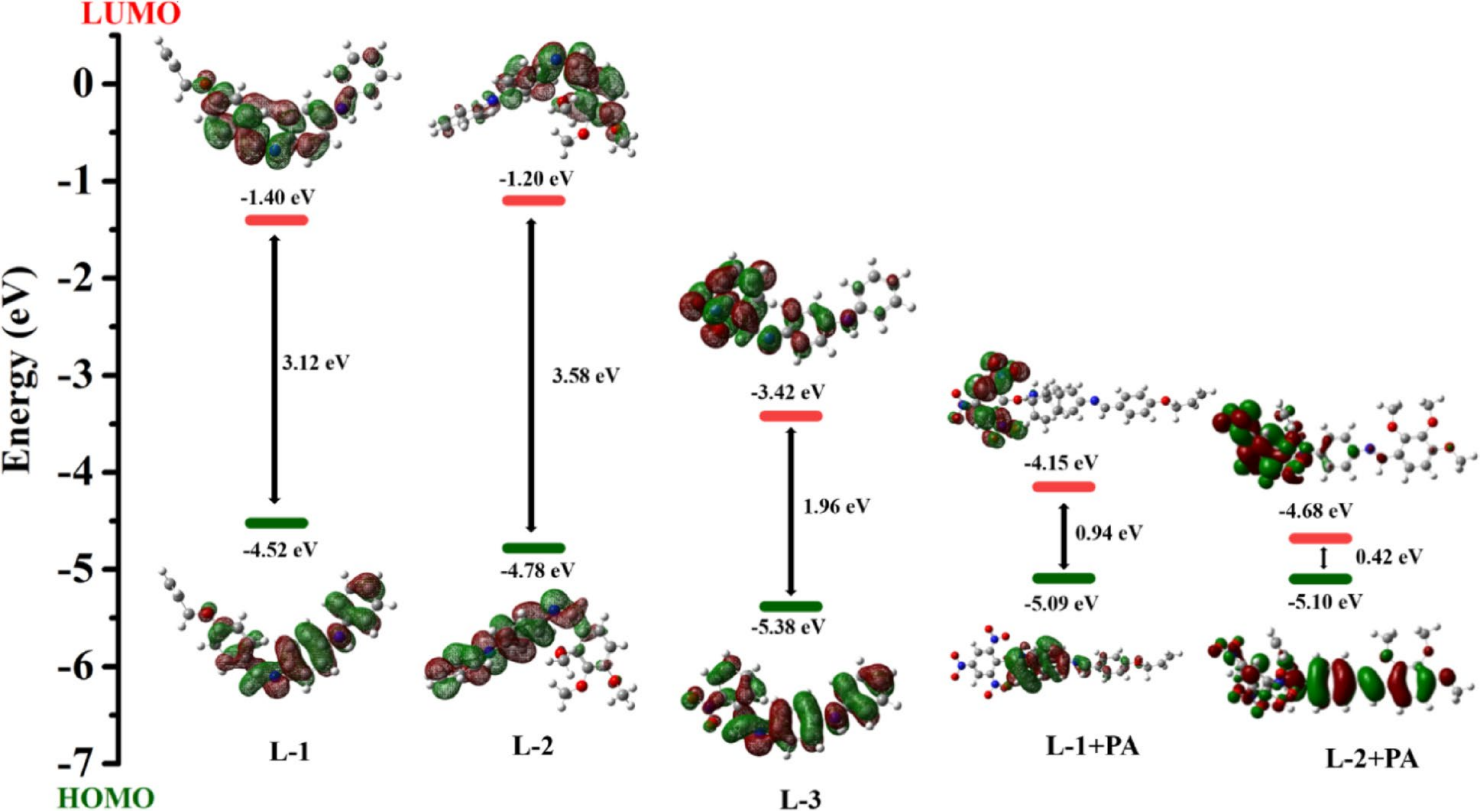
The orbital contribution of the probes **L1**, **L2**, **L-3**, **L1 + PA**, and **L2 + PA**

A Frontier Molecular Orbital (FMO) analysis provided further insights into the internal charge transfer (ICT) process that occurs upon the introduction of PA to the probes. Notably, in these probes, the Highest Occupied Molecular Orbital (HOMO) was primarily localized on the benzylidene unit, while the Lowest Unoccupied Molecular Orbital (LUMO) was partially distributed across both the benzylidene and aldehyde units. Following the addition of PA, a significant localization of electron density was observed in the benzylidene moiety for **L-1**. In contrast, for **L-2**, the electron density in the HOMO was more widely distributed, with the LUMO showing dispersion across the entire PA molecule. Furthermore, the LUMO value of L-3 was considerably lower, indicating that this moiety is electron-deficient and incapable of donate electrons to PA. The probes exhibited a higher LUMO, making them capable of donating electrons to PA through electrostatic interactions. The energy gaps between the HOMO and LUMO for **L-1** and **L-2** were found to be 3.12 eV and 3.58 eV, shown in Fig. 10. After the addition of PA, the HOMO − LUMO gaps reduced to 0.94 eV and 0.42 eV, respectively, leading to a bathochromic shift (redshift) in the UV-visible spectrum and indicating the formation of a more stable complex between **L-1**, **L-2**, and **PA**.

## Analytical application of L-1 and L-2

### Solid and solution state detection

**Fig. 11:**
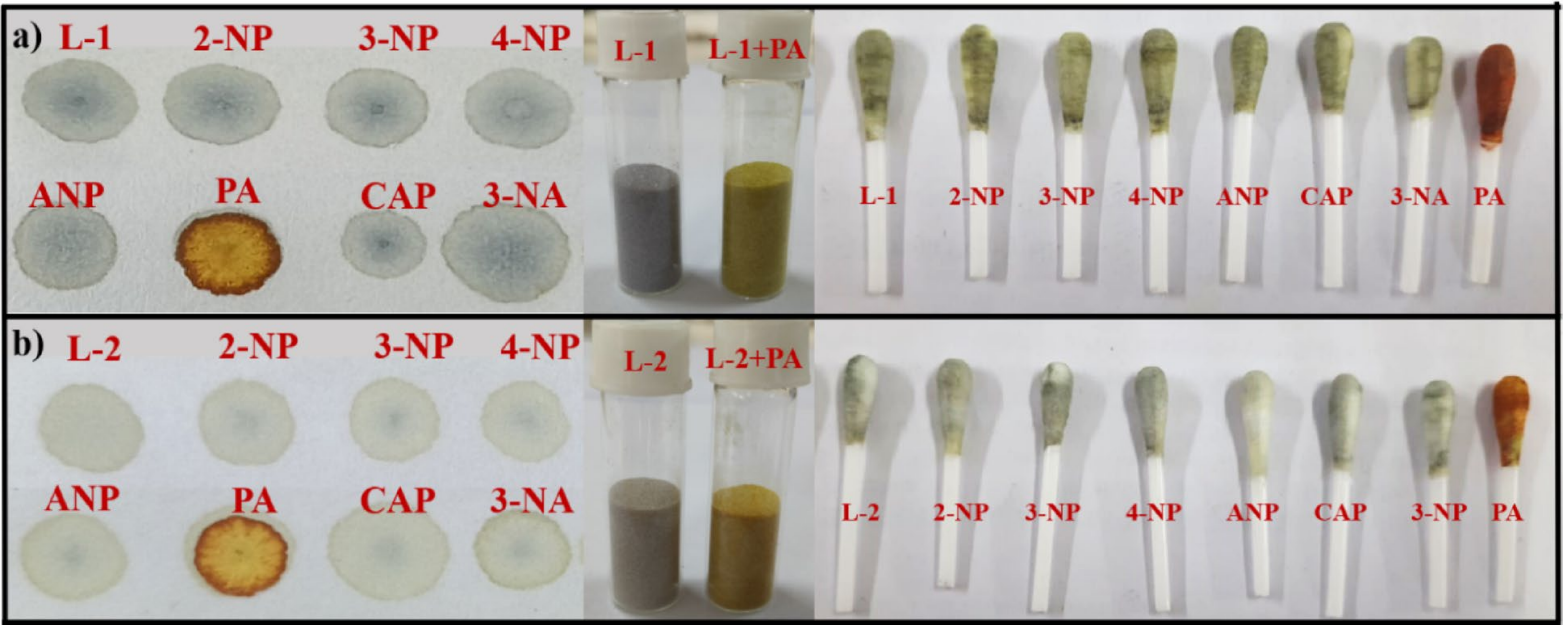
The test strip, silica gel, and cotton swab with other nitroaromatics

**Fig. 12:**
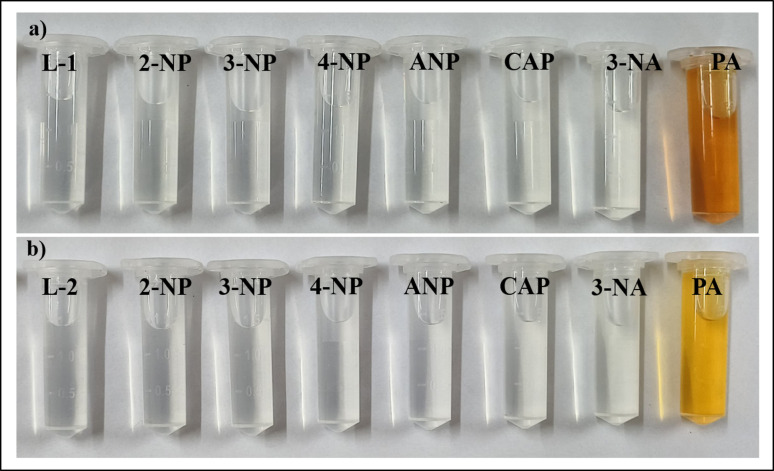
Normal light colour change of probes in CH_3_CN solution

Due to its various uses and widespread applications, PA can contaminate clothing, equipment, or the human body. To improve the practical applications for detecting PA, tests were performed using probes. Test strips were prepared by applying 10 µL of 1 × 10^− 3^ M solutions of the probes in CH_3_CN separately. After the solvent evaporated, 2 µL of a 1 × 10^− 3^ M PA solution was placed onto the treated filter paper using a glass capillary tube. The observed colour change is shown in Fig. 11. A similar colour change of the probe after the introduction of PA was also monitored in solution, as depicted in Fig. 12.

### Smartphone-assisted PA detection

**Fig. 13:**
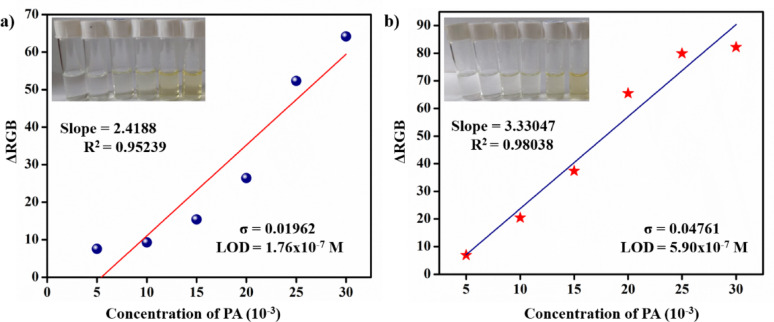
RGB plot of **a** L-1 and **b** L-2 towards the detection of PA

The RGB smartphone technique has been utilized as an analytical tool for detecting PA using **L-1** and **L-2.** Various concentrations of PA (1 × 10⁻³ M) were added to the probe solution in CH₃CN, leading to observable colour changes. Images were captured using the RGB tool, which is available as an app for all types of Android devices. The RGB values were extracted from the tool based on colour intensity. A plot was created with the concentration of PA on the X-axis and ΔRGB on the Y-axis (see Fig. 13). This allowed for the determination of the limit of detection (LOD) for **L-1** (5.90 × 10⁻⁷ M) and **L-2** (1.76 × 10⁻⁷ M). The LOD was calculated similarly to how it is derived from the slope and intercept of the linear plot of absorbance versus concentration (see Fig. 4). Thus, this smartphone technique facilitates practical and quantitative detection of the analyte.

### Identification of PA in the matchstick powder sample

**Fig. 14:**
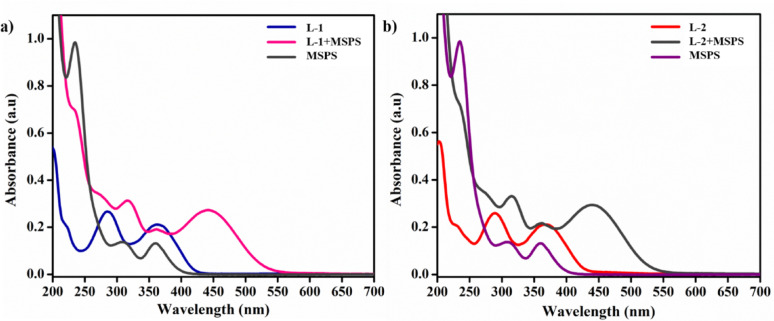
The observed UV spectral changes of the probes with MSPS

To investigate the real-time application of the probes for PA, matchstick powder was chosen as the analyte. A sample weighing approximately 500 mg of matchstick head powder was thoroughly ground using a mortar and pestle. This powder was then mixed with 10 mL of acetonitrile solution and sonicated for about 10 min. The solution was extracted following the protocol detailed in references [35–37] and subsequently analyzed using UV-Vis absorption spectroscopy. The UV spectrum was recorded by adding 20 µL of the matchstick powder solution to the probes, without the addition of PA. A sudden shift in the λ_max_ of the probes was observed, as shown in Fig. 14.

To conclude all the newly developed probes are compared with the already available probes in the literature and are given the Table 2.

**Table 2:** Comparative study with previously reported works of literature

Refs.	Receptor	LOD	Target Analyte	References
[1]	Pyridine-pyrazole based	1.22 × 10^− 4^	PA, Al^3+^	[38]
[2]	ZnSe quantum dots	1.24 × 10^− 5^	PA	[39]
[3]	Ca (II)-MOF	1.90 × 10^− 5^	PA	[40]
[4]	Quinoxaline based derivatives	1.37 × 10^− 6^	PA	[41]
[5]	Allyloxy benzylidene-amino based	4.37 × 10^− 7^ M	PA	Present work
	Trimethoxy benzylidene-amino based	4.56 × 10^− 6^ M	PA	

## Conclusion

In this study, we developed and synthesized benzylidene-amine appended N-phenylaniline derivatives and characterized them. Probe **L3** did not exhibit significant detection capabilities for PA, whereas probes **L-1** and **L-2** demonstrated both sensitivity and selectivity toward **PA**. This enhanced response is attributed to the presence of electron-rich substituents, which increase the basicity of the -NH unit, facilitating the formation of **L-1 + PA** and **L-2 + PA** complexes. Since **PA** is more electron-deficient compared to other nitroaromatics, this interaction is particularly significant. The limits of detection for **L-1** and **L-2** were calculated to be 4.37 × 10^− 7^ M and 4.56 × 10^− 6^ M for **PA**, respectively. Additionally, the formation of **L-1 + PA** and **L-2 + PA** was confirmed through ^1^H NMR titration. Density functional theory studies were supported by the orbital composition distributions of the HOMO and LUMO. Furthermore, the reported probes can effectively sense PA in real-world samples, such as matchstick powder, reinforcing their practical applicability.

## Supplementary Information

Below is the link to the electronic supplementary material.

Supplementary Material 1

## Data Availability

No datasets were generated or analysed during the current study.
