# Supplementary material for: A benzylidene-amine scaffold as a colourimetric sensor for picric acid: computational studies and real-time applications using matchstick head powder
Source: BMC Chem. 2025 Nov 21;19(1):310. doi: 10.1186/s13065-025-01670-4 (PMC12639742; doi:10.1186/s13065-025-01670-4)
Supplement: Supplementary file 1 — Supplementary Material 1 [file 13065_2025_1670_MOESM1_ESM.docx]

**Supplementary Material**

**A Benzylidene-amine scaffold as a picric acid colourimetric sensor: Computational studies and real-time applications on matchstick head powder**

**Viswanathan Hemalatha, Sundaramoorthy Sarveswari and Vijayaparthasarathi Vijayakumar***

*Department of Chemistry, School of Advanced Sciences, Vellore Institute of Technology, Vellore 632014, Tamil Nadu, India*

***Corresponding author:** vvijayakumar@vit.ac.in


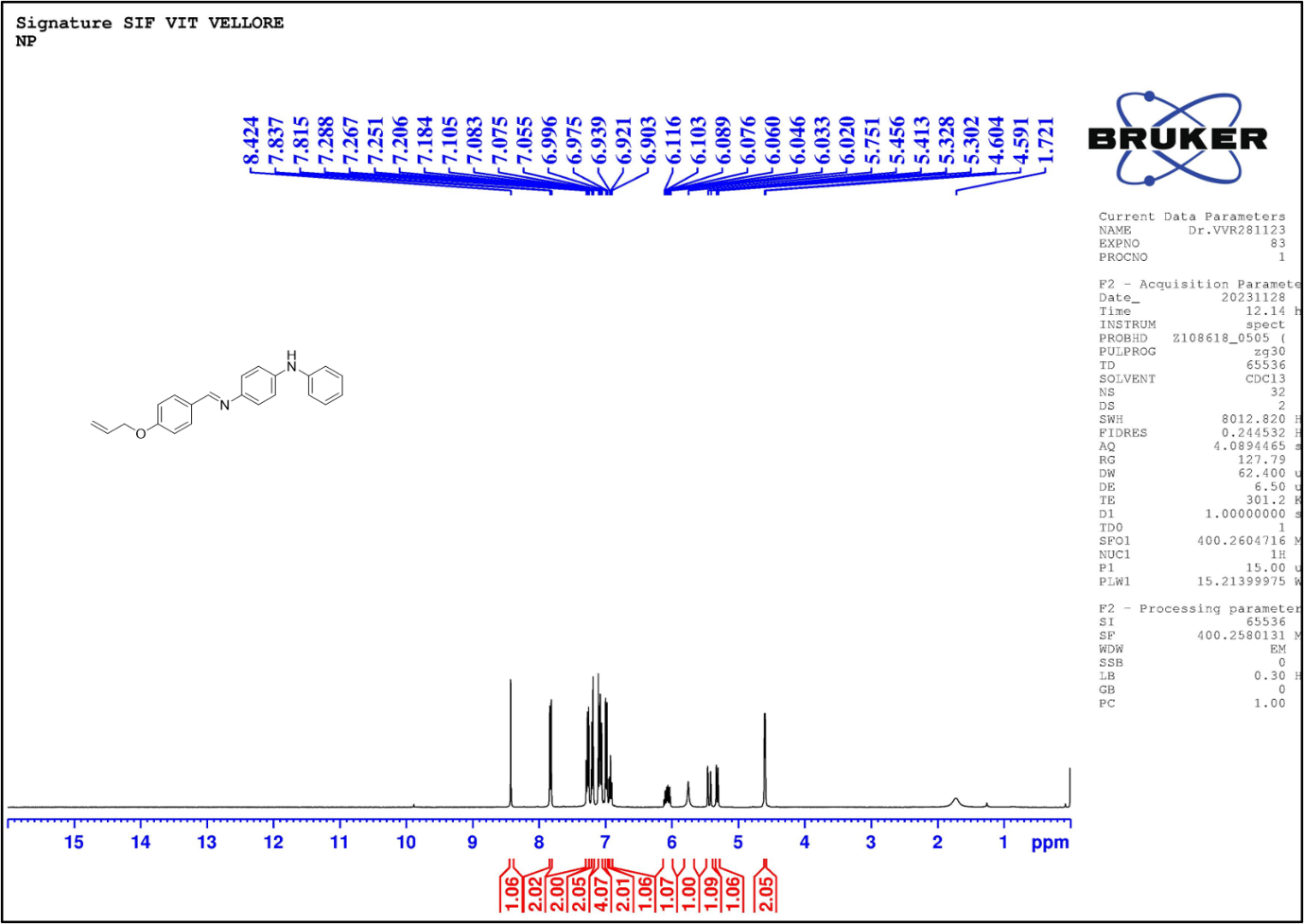


**Fig. S1.** ^1^H-NMR spectrum of **L-1** in CDCl_3_ at 25 ^o^C


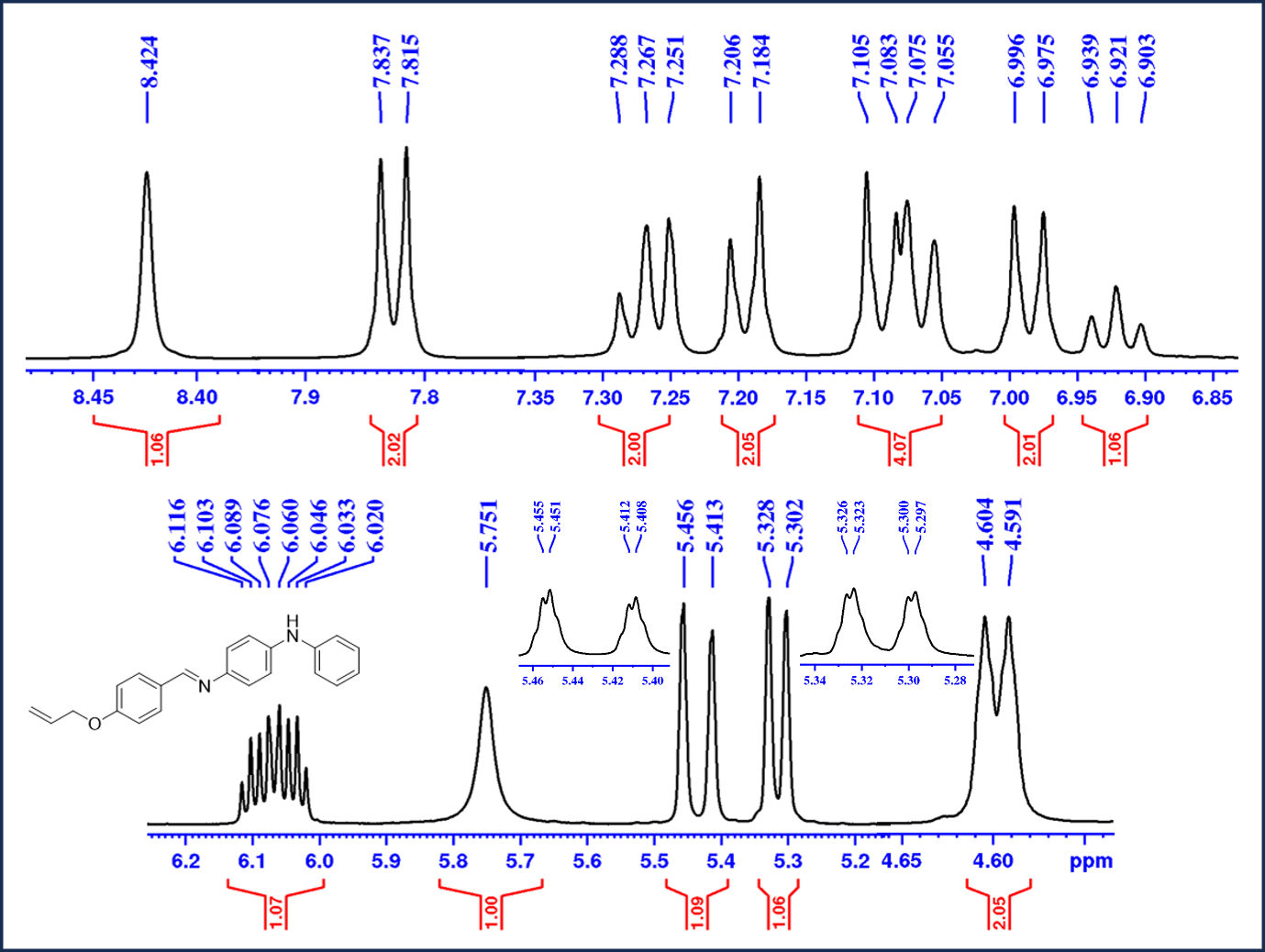


**Fig. S2.** ^1^H-NMR expanded spectrum of **L-1** in CDCl_3_ 25 ^o^C


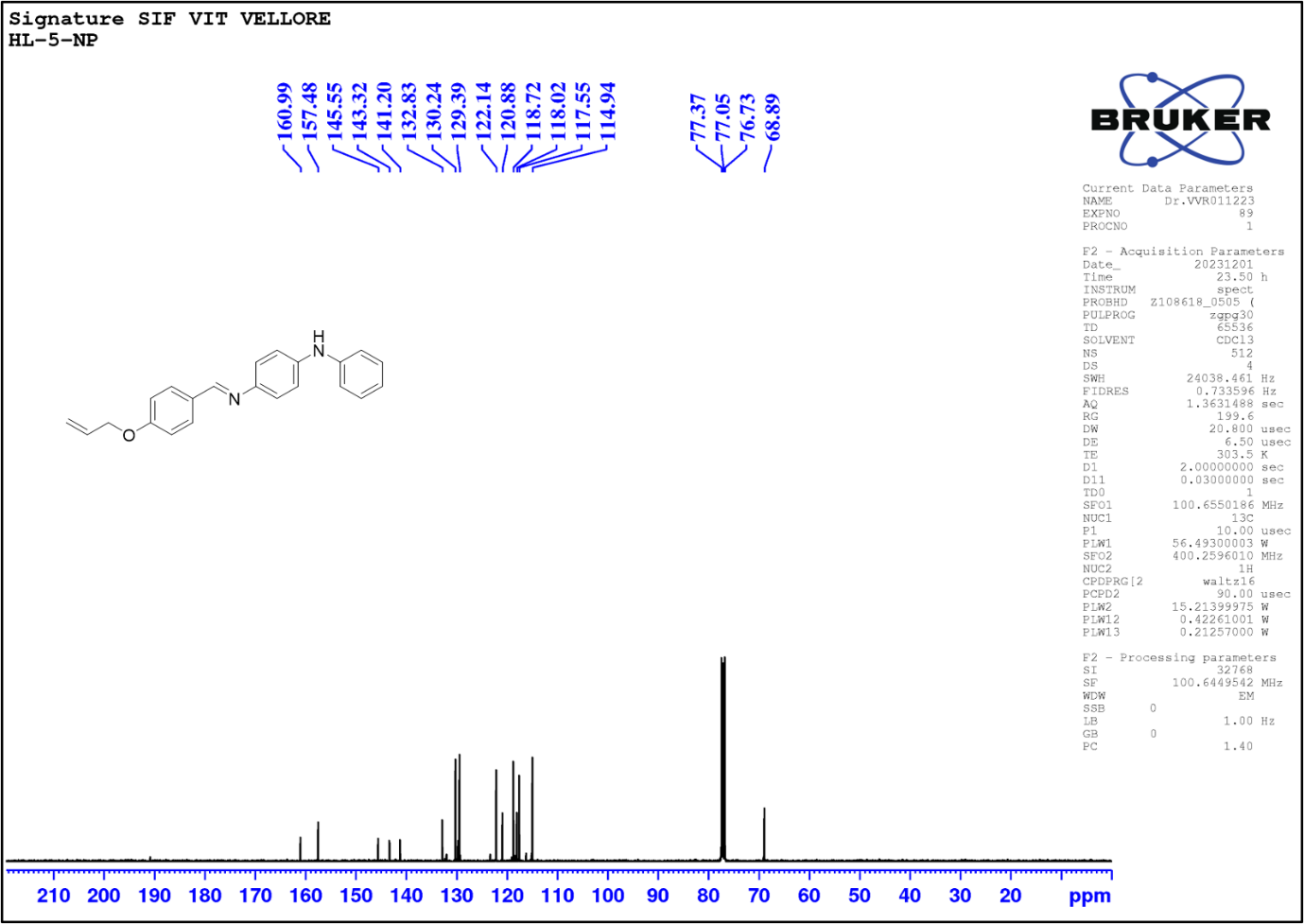


**Fig. S3.** ^13^C-NMR spectrum of **L-1** in CDCl_3_ 25 ^o^C


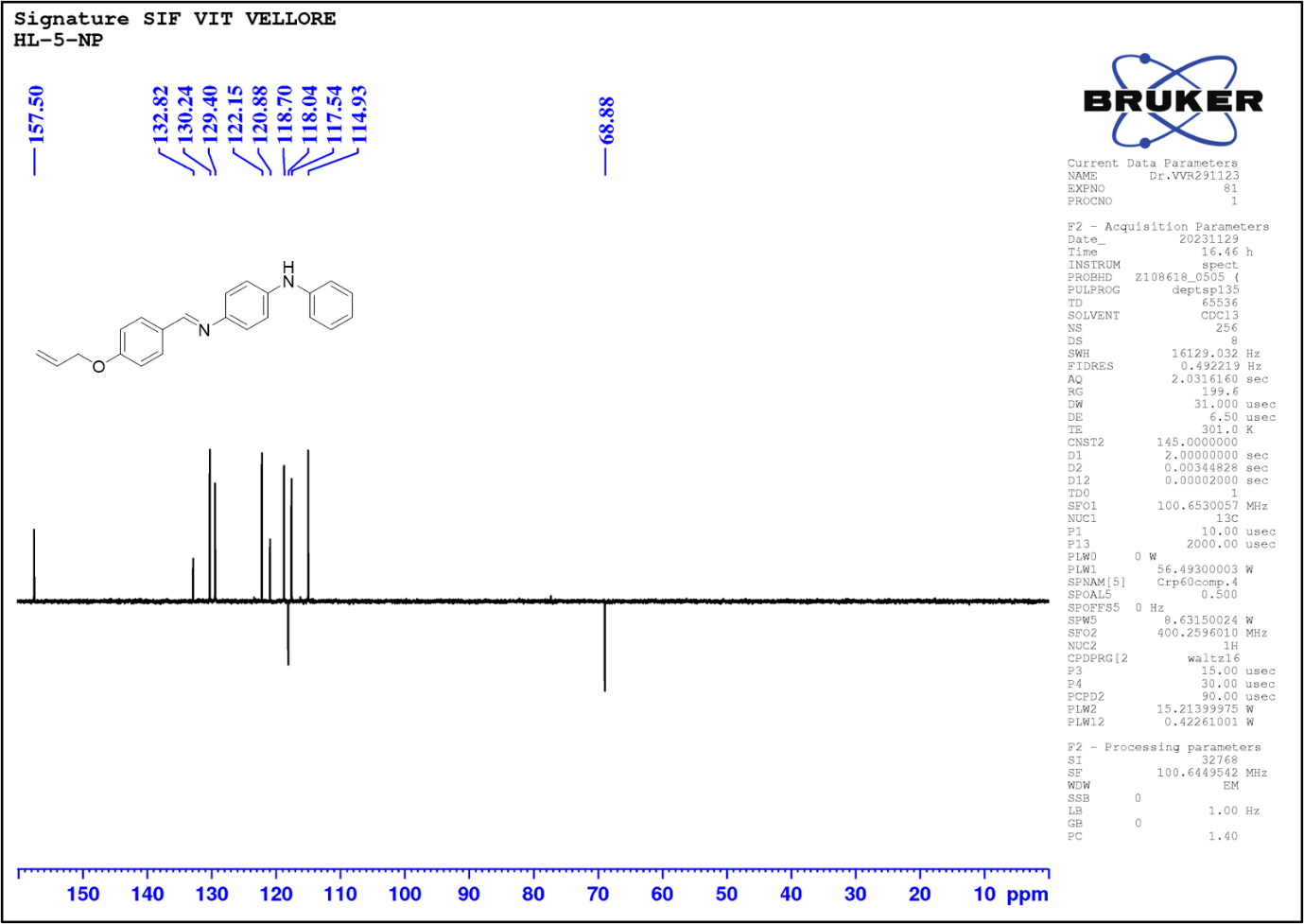


**Fig. S4.** DEPT-135 spectrum of **L-1** in CDCl_3_ 25 ^o^C


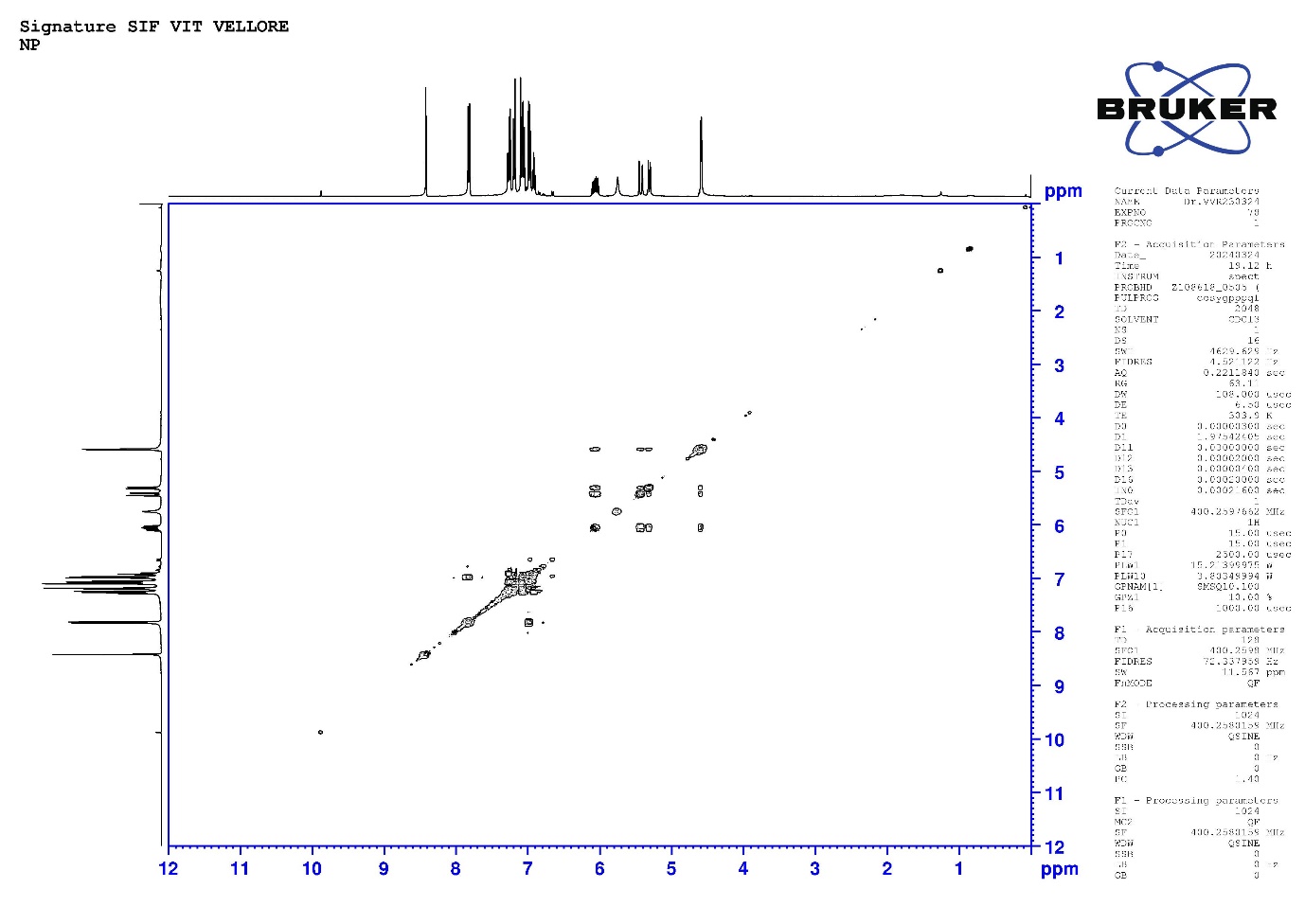


**Fig. S5.** ^1^H-^1^H COSY spectrum of **L-1** in CDCl_3_ at 25 ^o^C


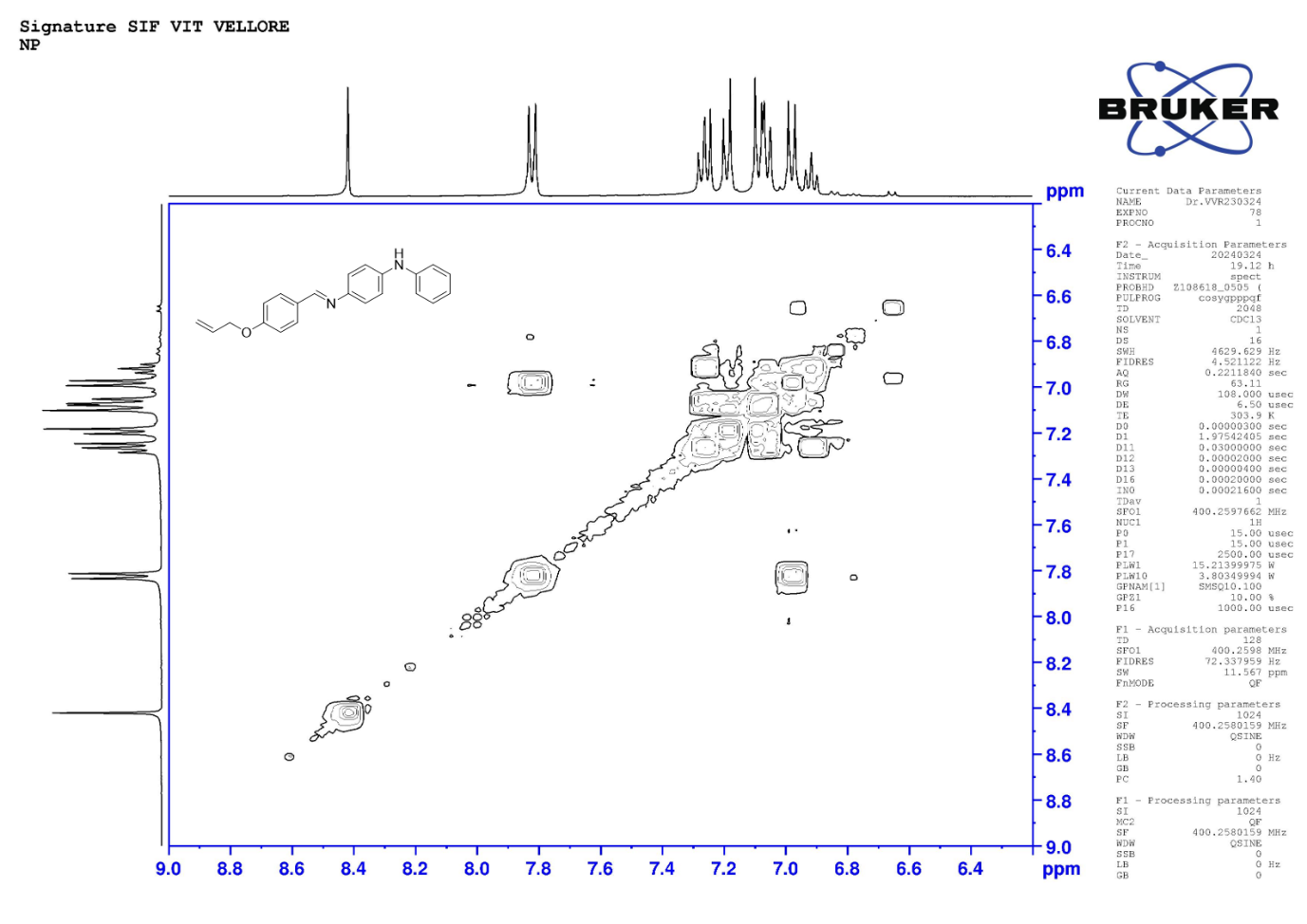


**Fig. S6.** ^1^H-^1^H COSY expanded spectrum of **L-1** in CDCl_3_ at 25 ^o^C


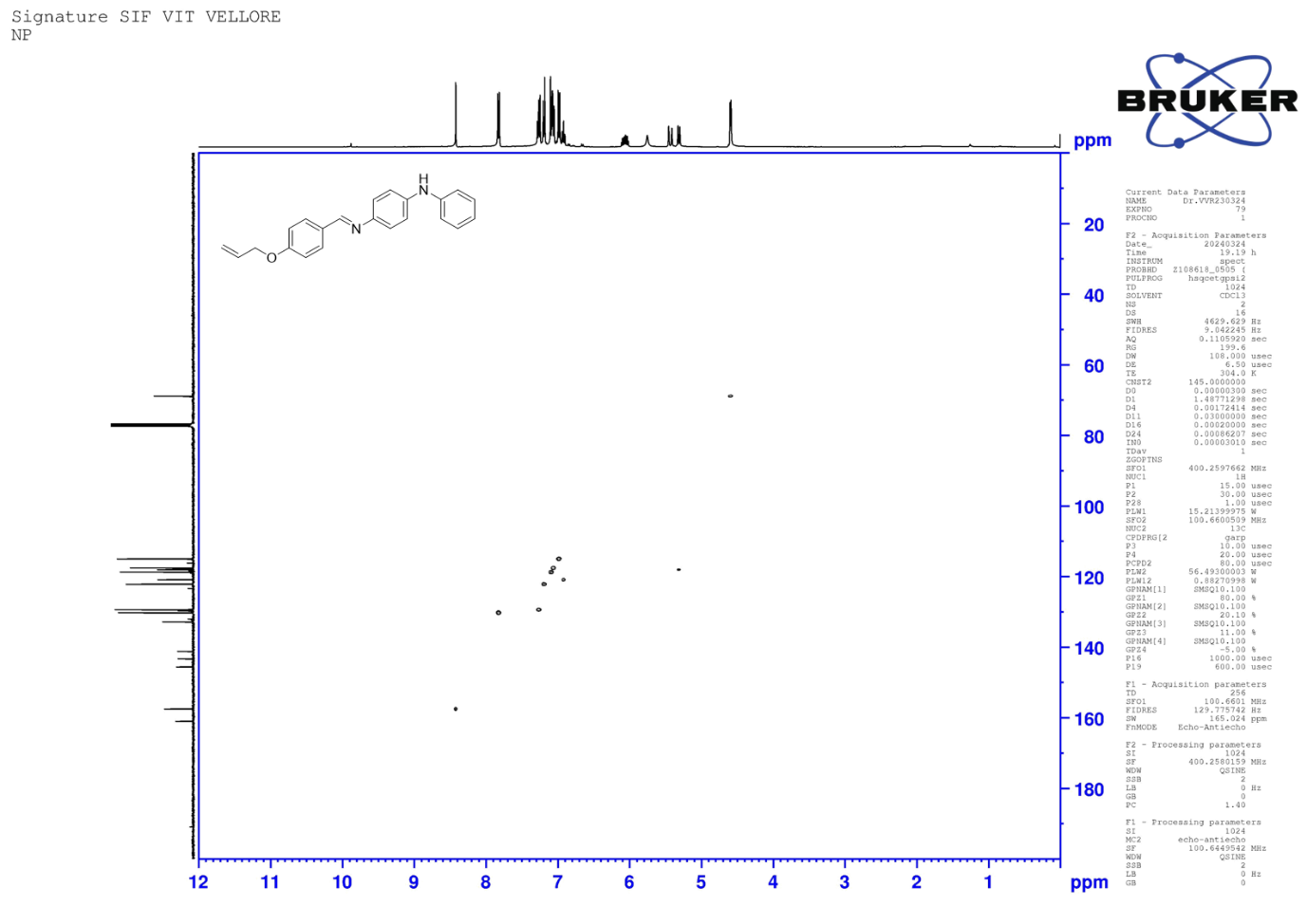


**Fig. S7.**  HSQC spectrum of **L-1** in CDCl_3_ at 25 ^o^C


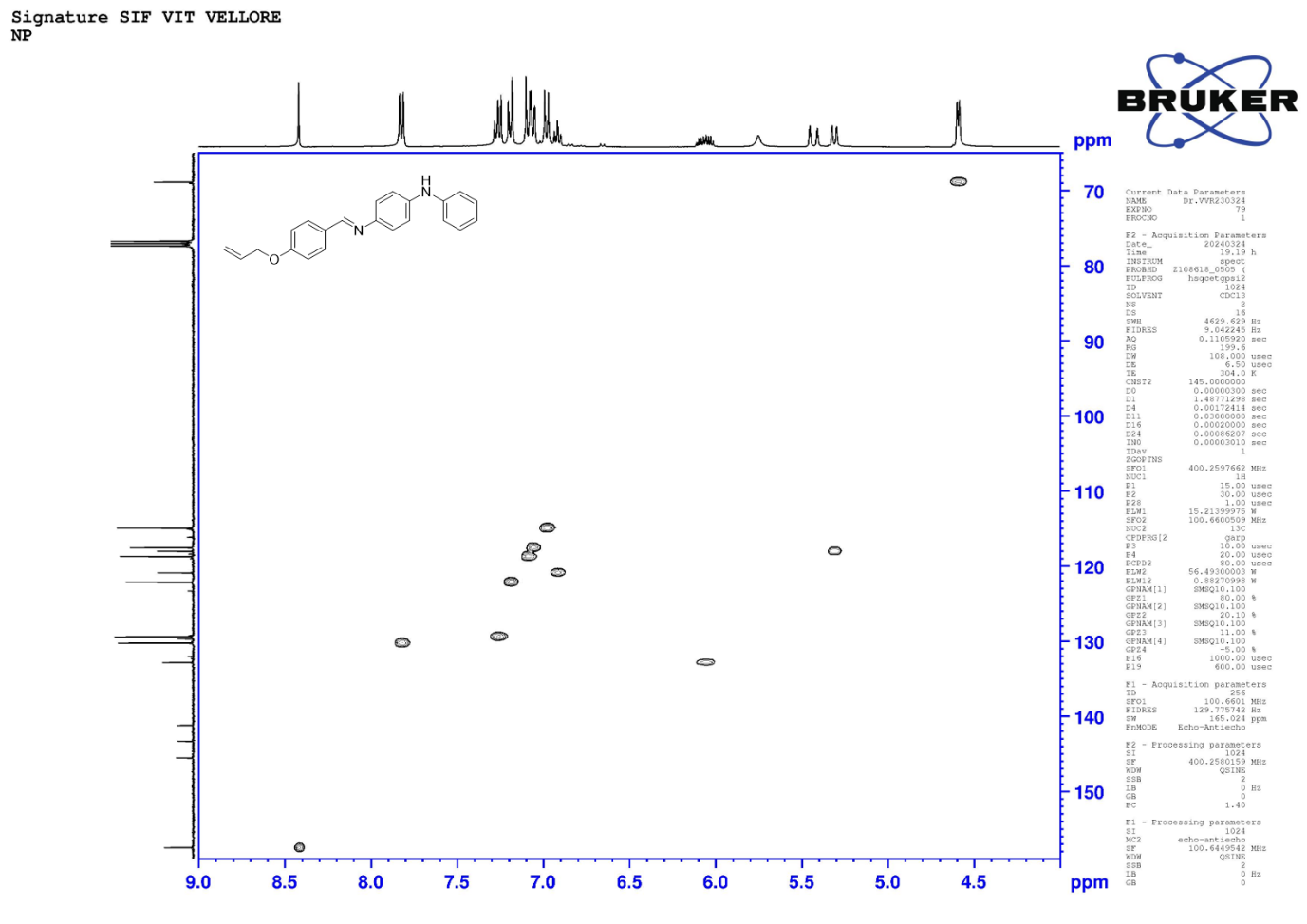


**Fig. S8.**  HSQC expanded spectrum of **L-1** in CDCl_3_ at 25 ^o^C


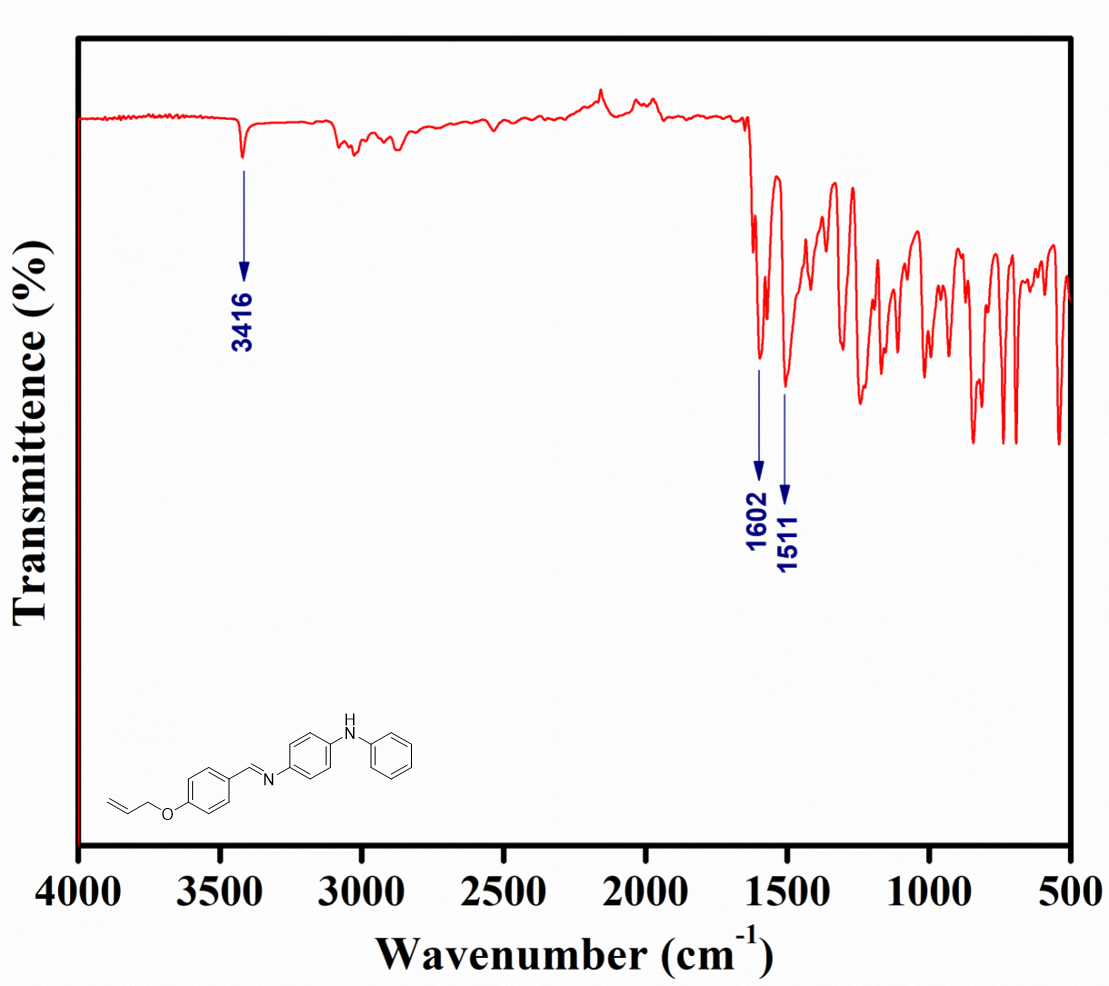


**Fig. S9.** FT-IR spectrum of **L-1**

**
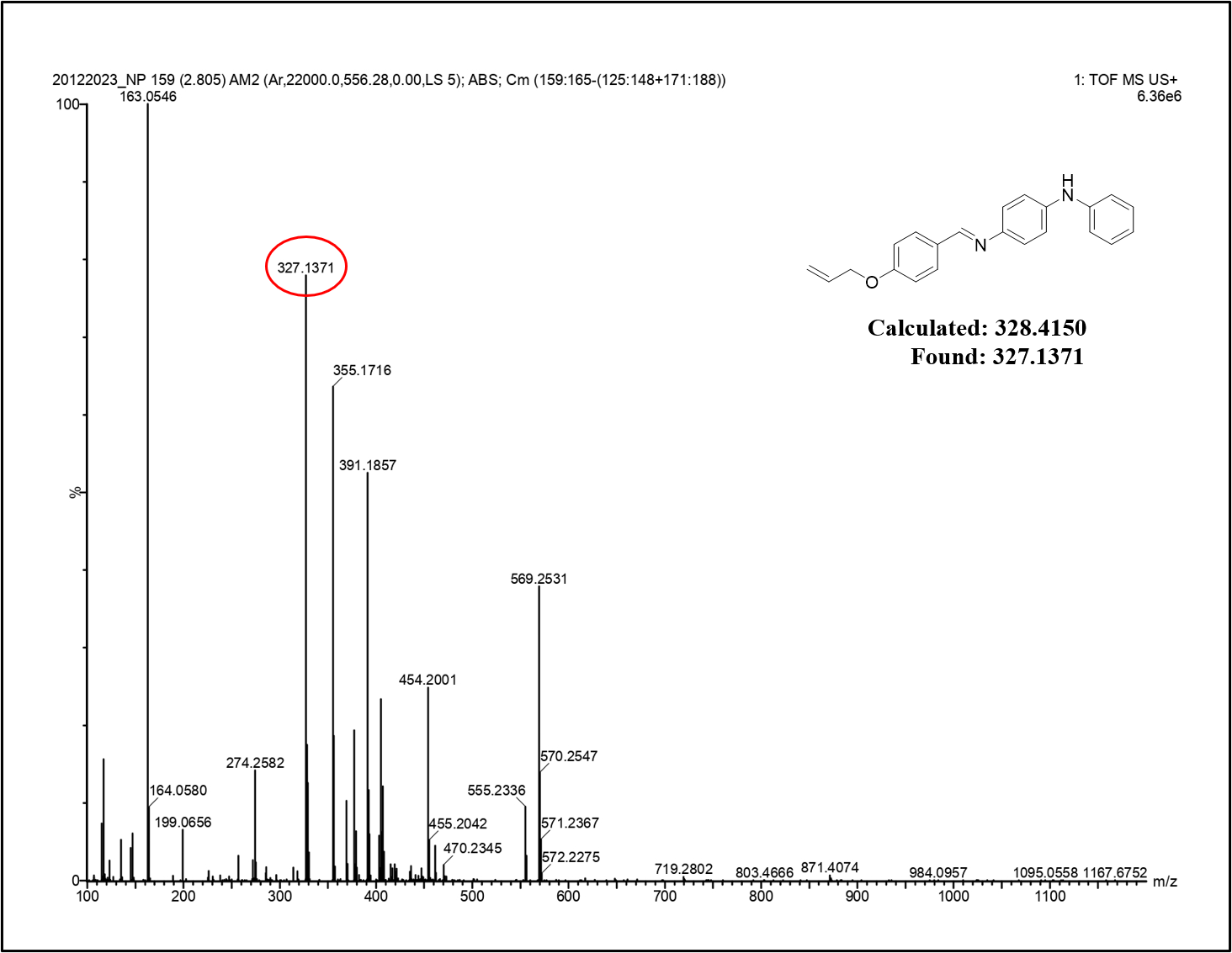
Fig. S10.** HR-Mass spectrum of **L-1**


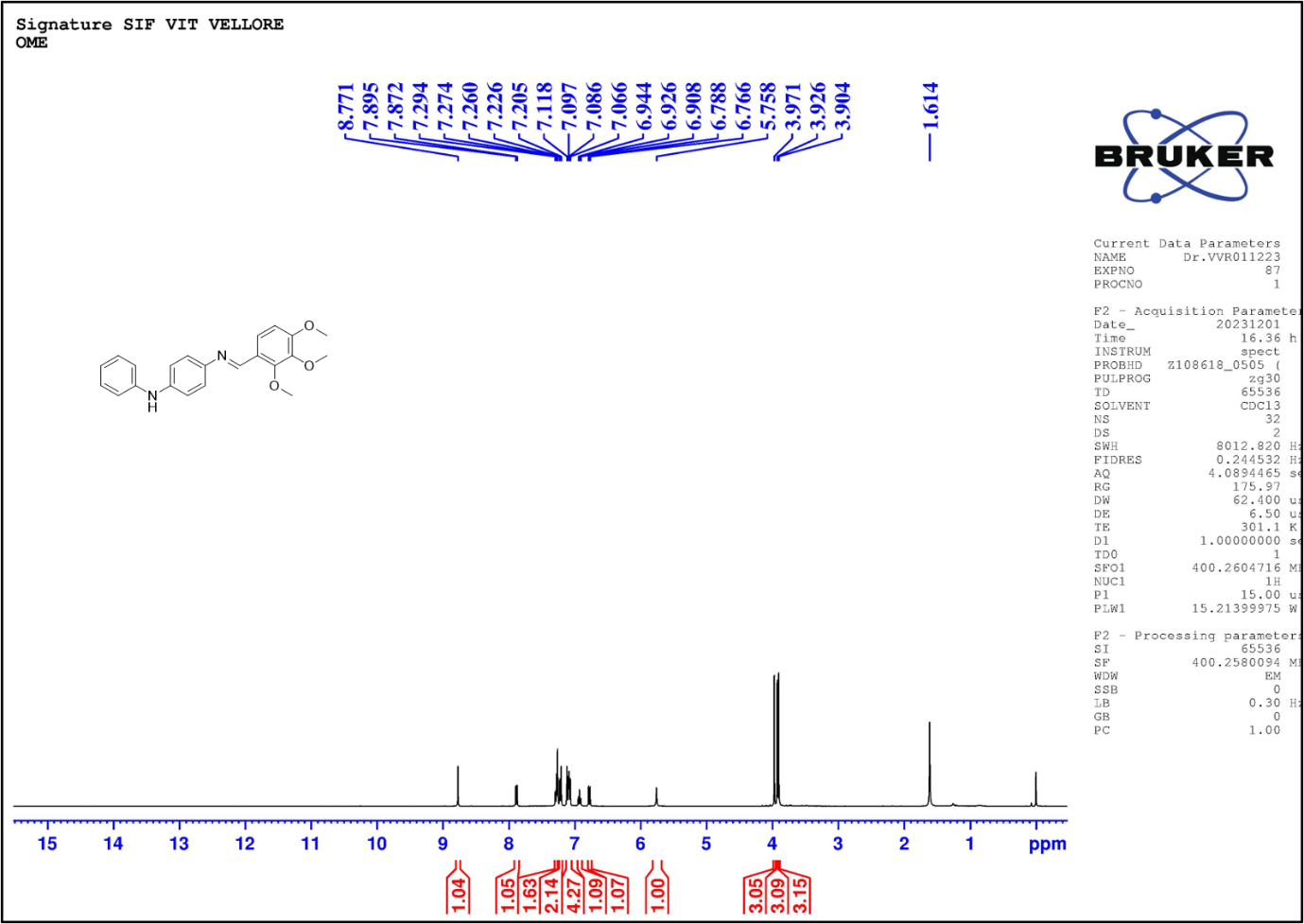


**Fig. S11.** ^1^H-NMR spectrum of **L-2** in CDCl_3_ at 25 ^o^C


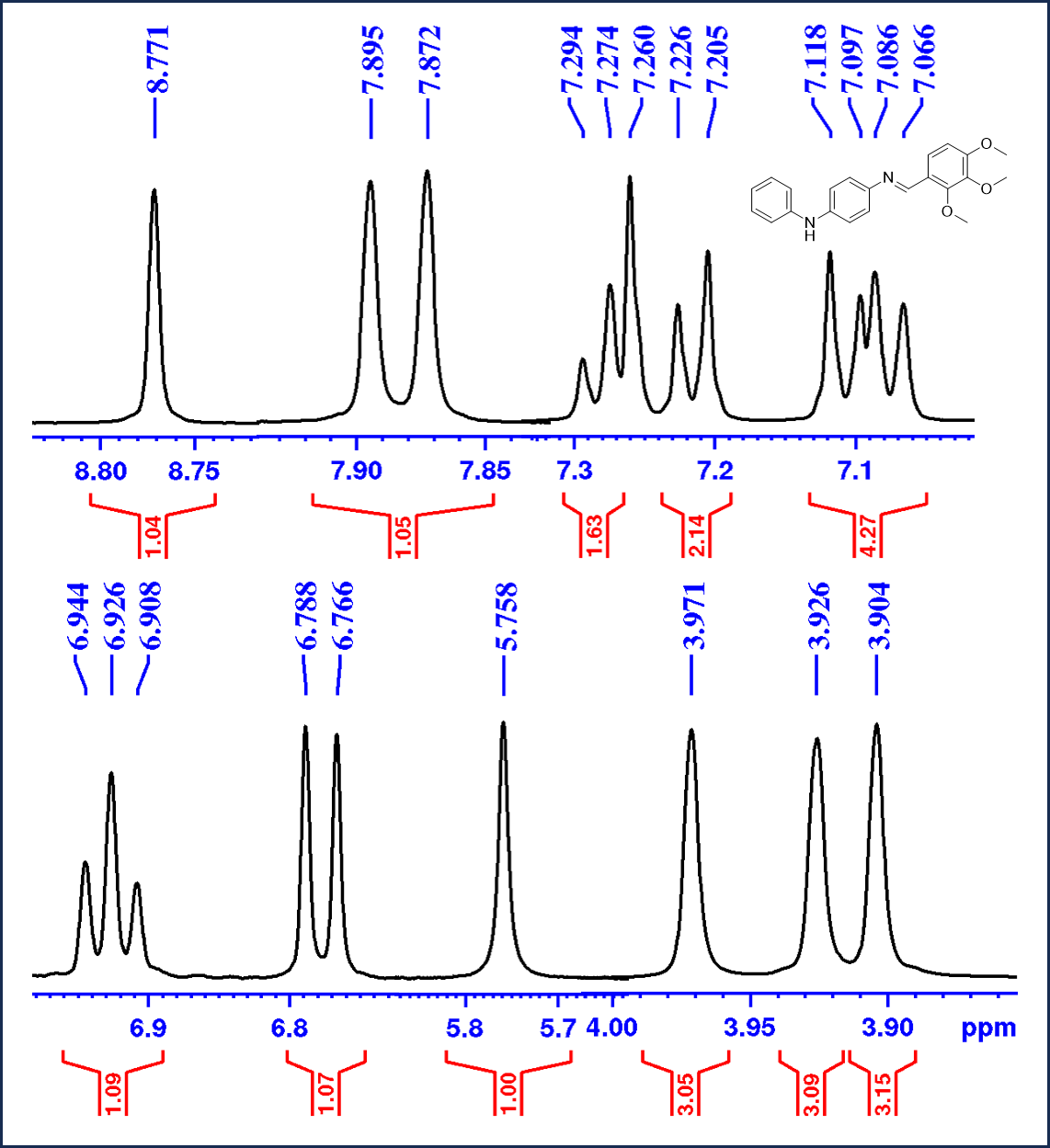
­

**Fig. S12.** ^1^H-NMR expanded spectrum of **L-2** in CDCl_3_ 25 ^o^C


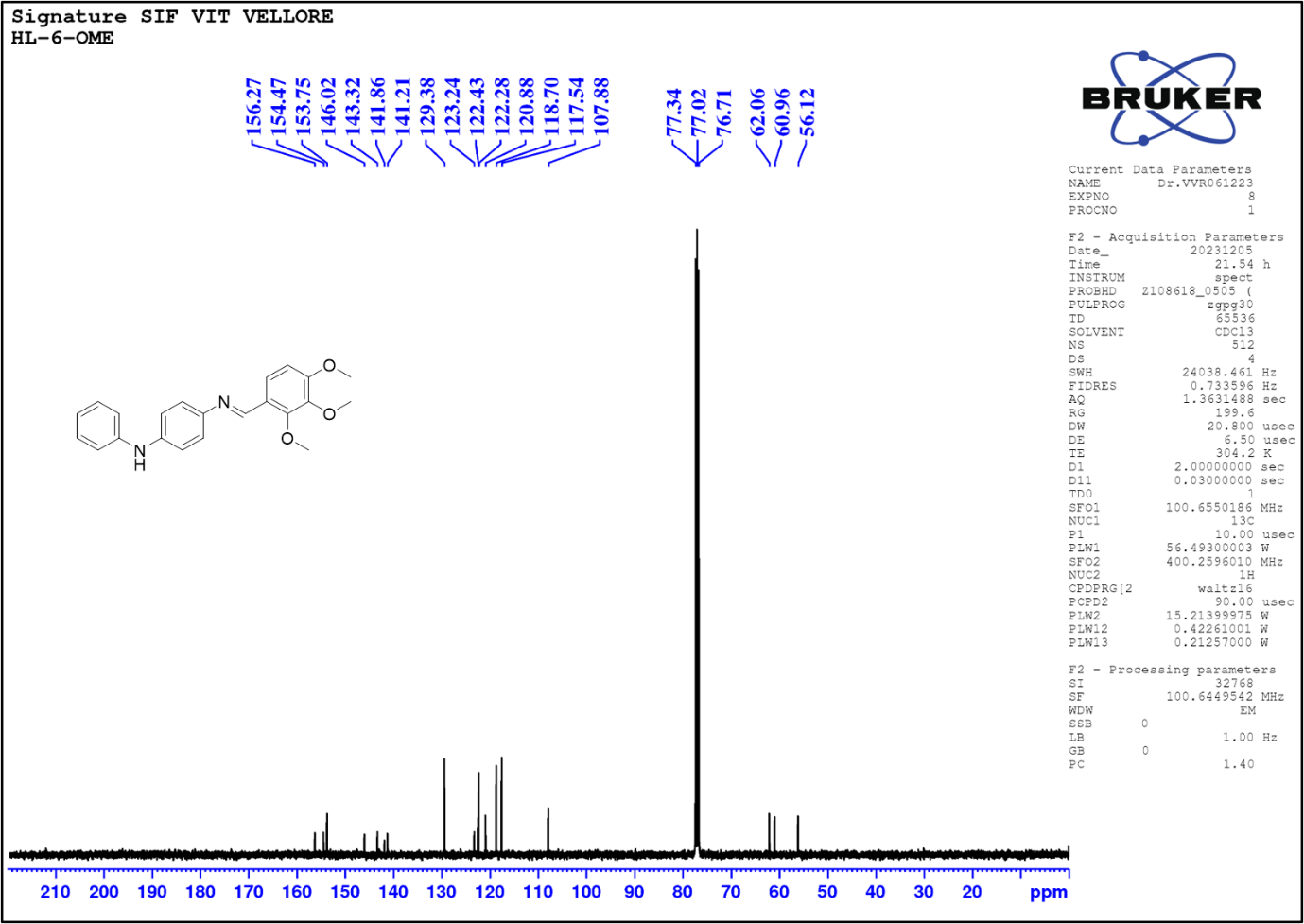


**Fig. S13.** ^13^C-NMR spectrum of **L-2** in CDCl_3_ 25 ^o^C


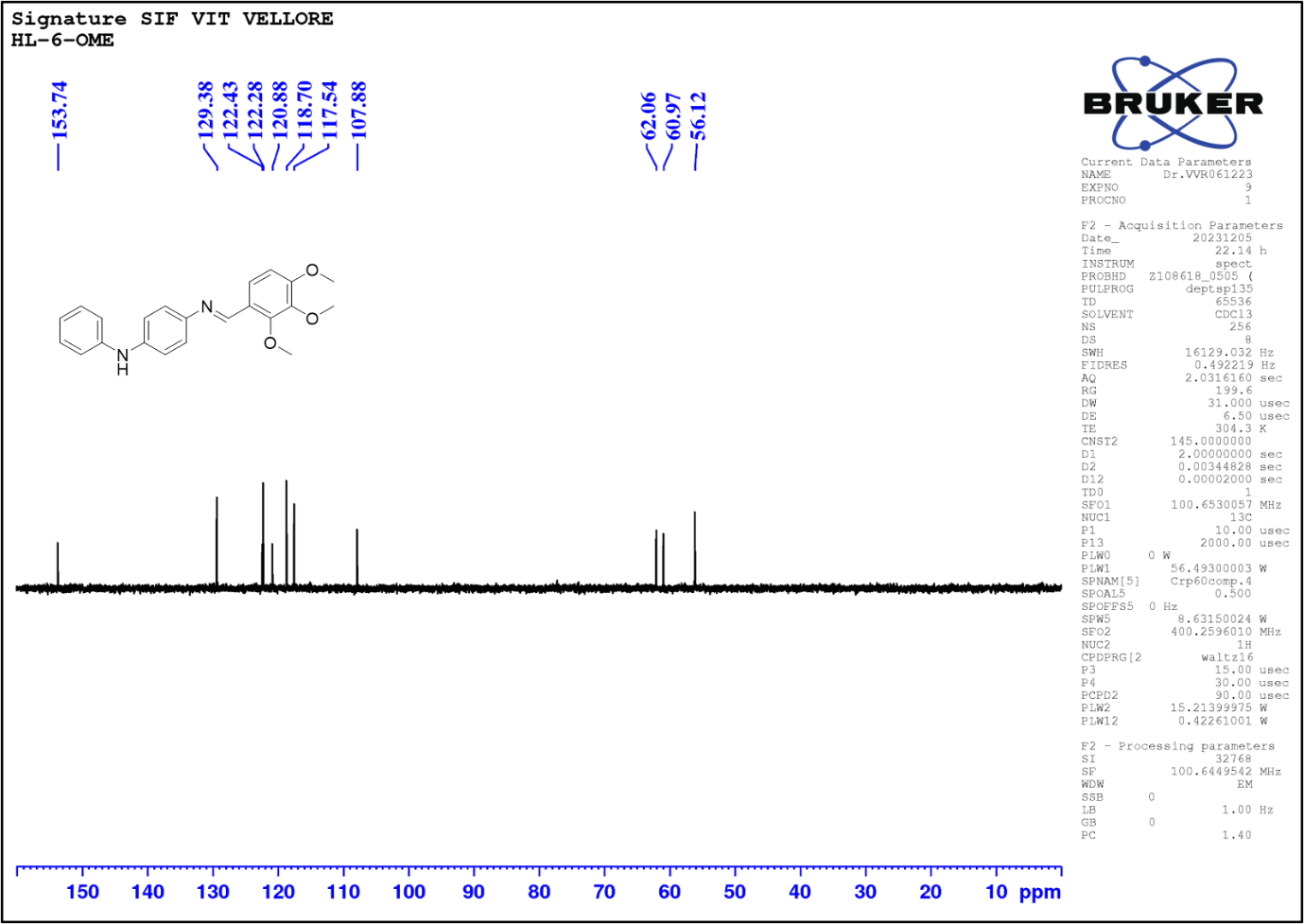


**Fig. S14.** DEPT-135 spectrum of **L-2** in CDCl_3_ 25 ^o^C


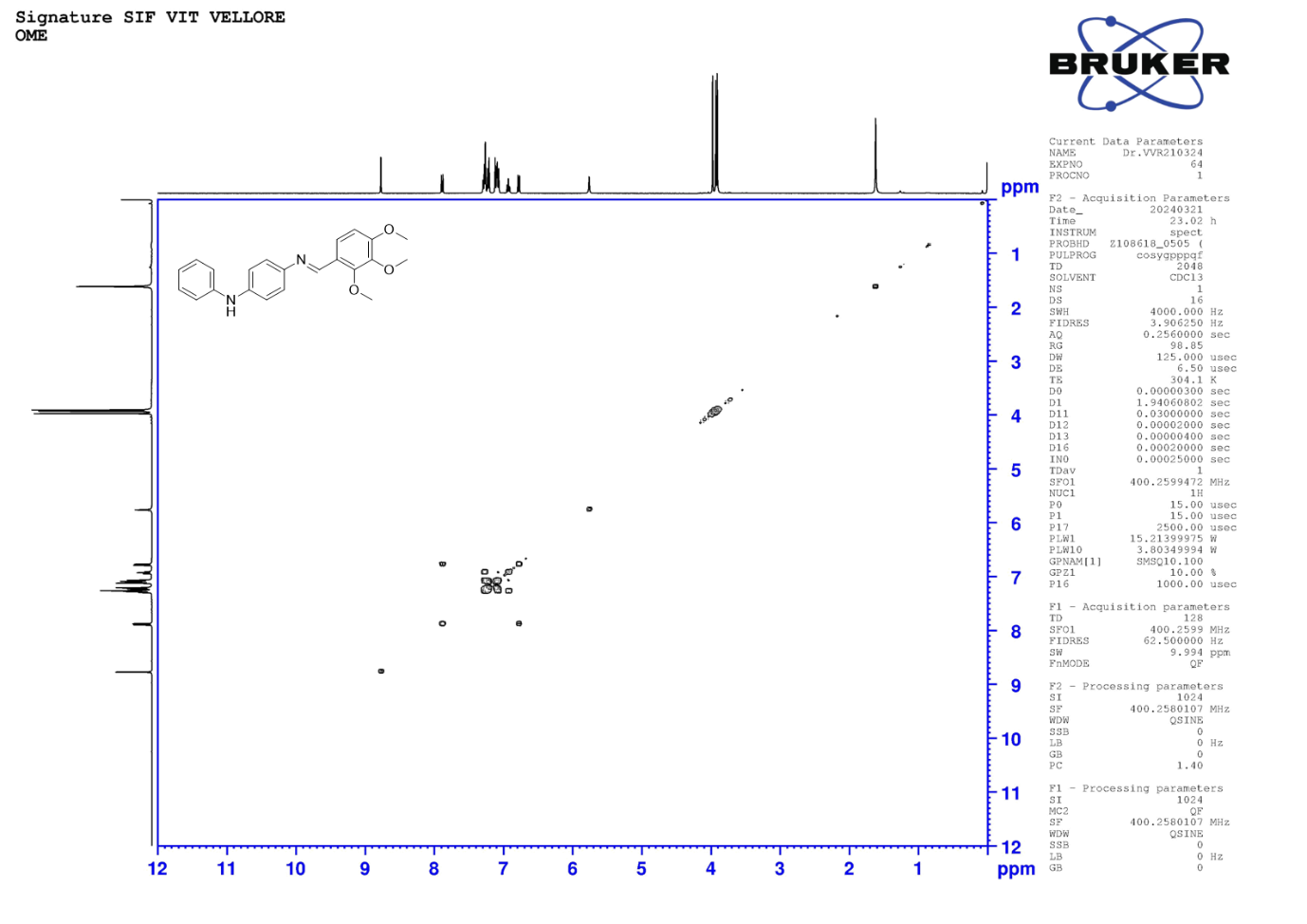


**Fig. S15.** ^1^H-^1^H COSY spectrum of **L-2** in CDCl_3_ at 25 ^o^C


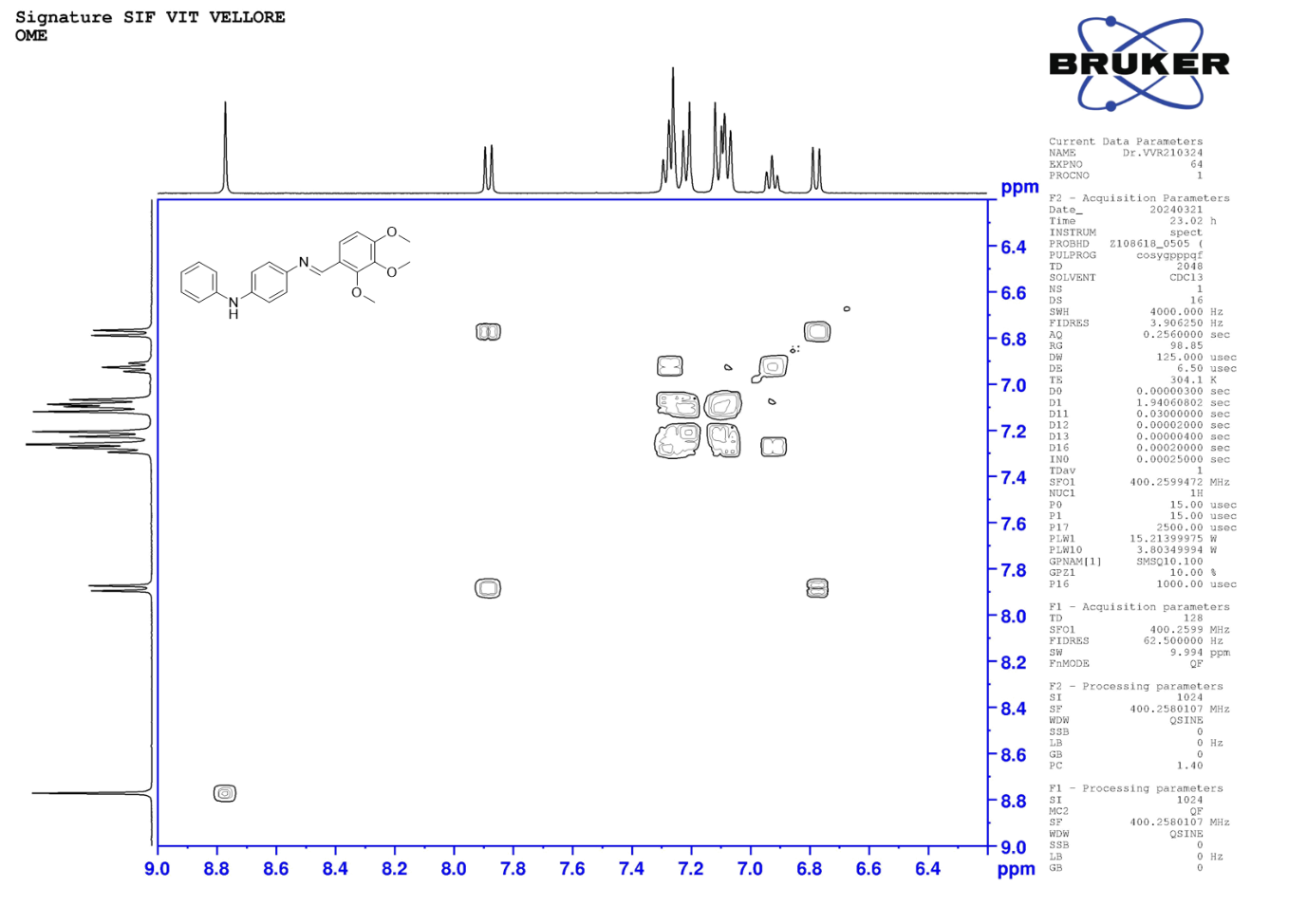


**Fig. S16.** ^1^H-^1^H COSY expanded spectrum of **L-2** in CDCl_3_ at 25 ^o^C


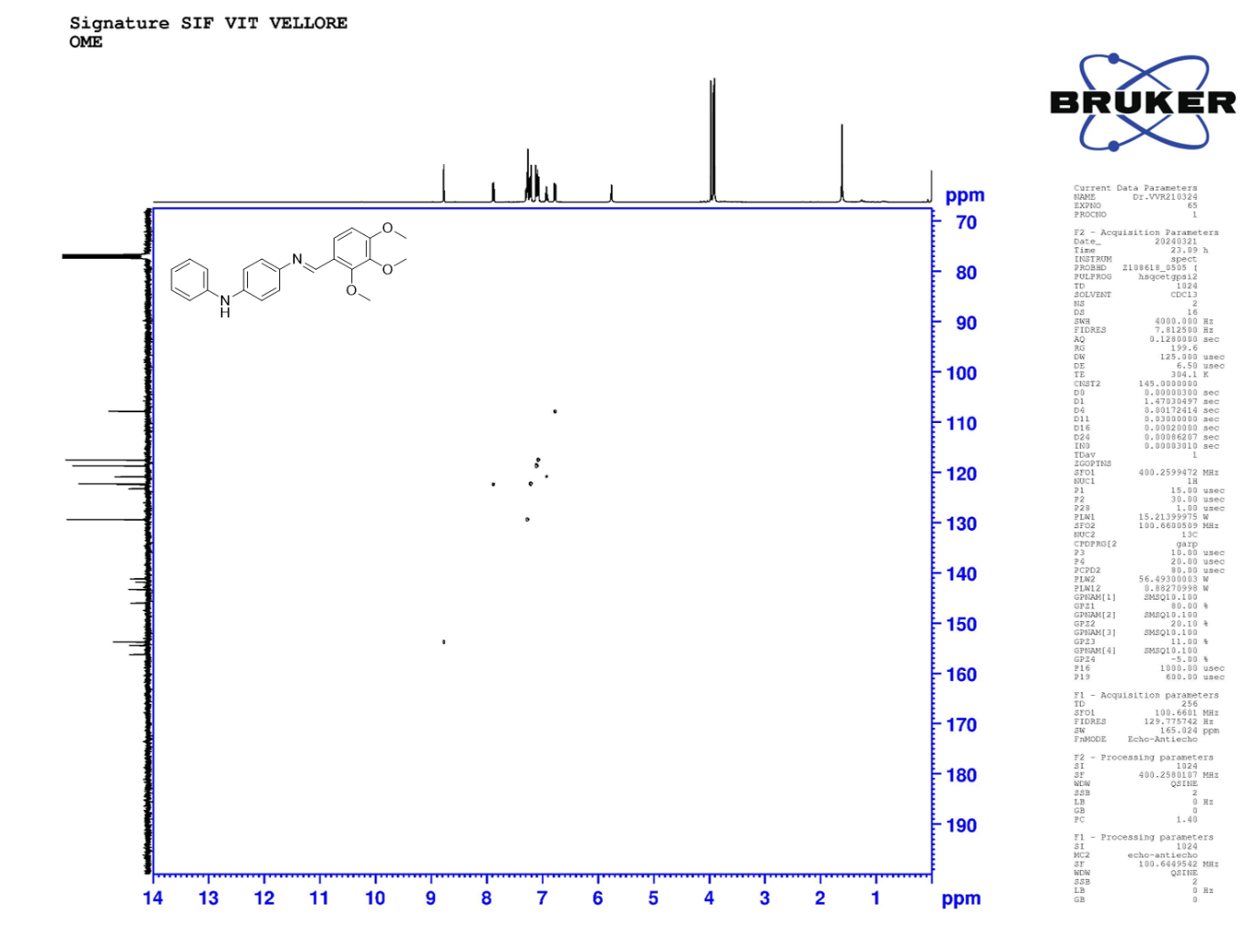


**Fig. S17.**  HSQC aromatic region spectrum of **L-2** in CDCl_3_ at 25 ^o^C

**
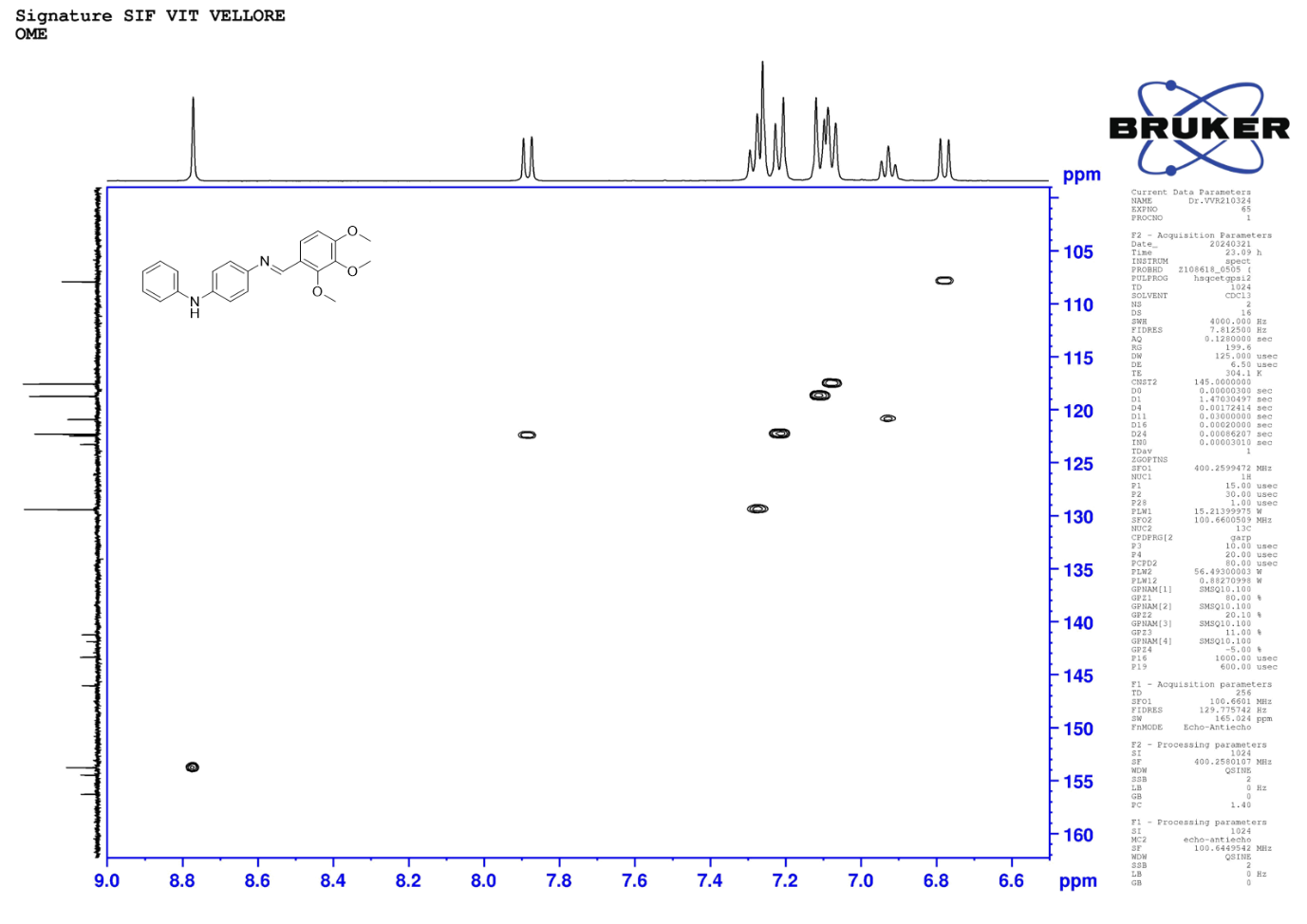
Fig. S18.**  HSQC expanded aromatic region spectrum of **L-2** in CDCl_3_ at 25 ^o^C


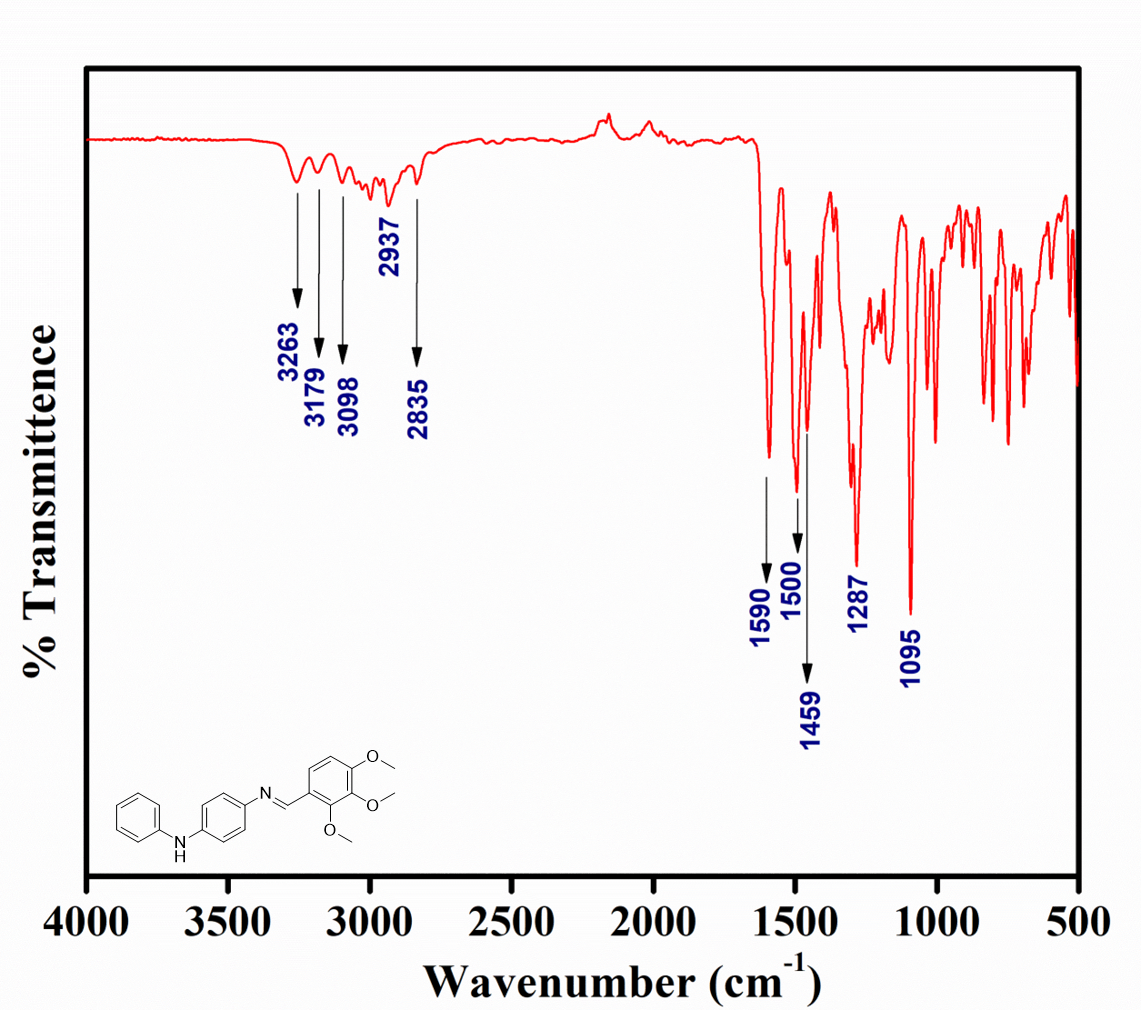


**Fig. S19.** FT-IR spectrum of **L-2**


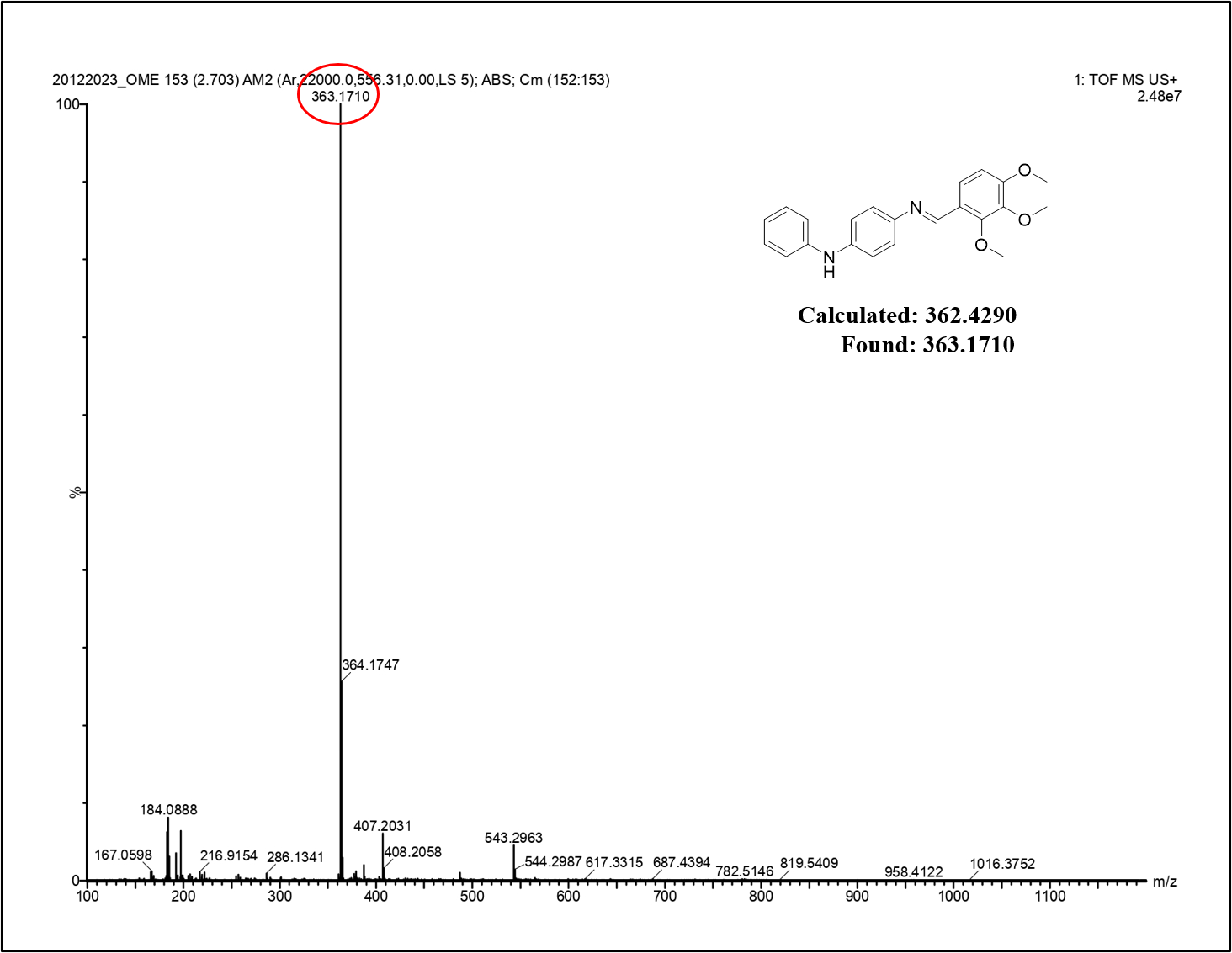


**Fig. S20.** HR-Mass spectrum of **L-2**

**
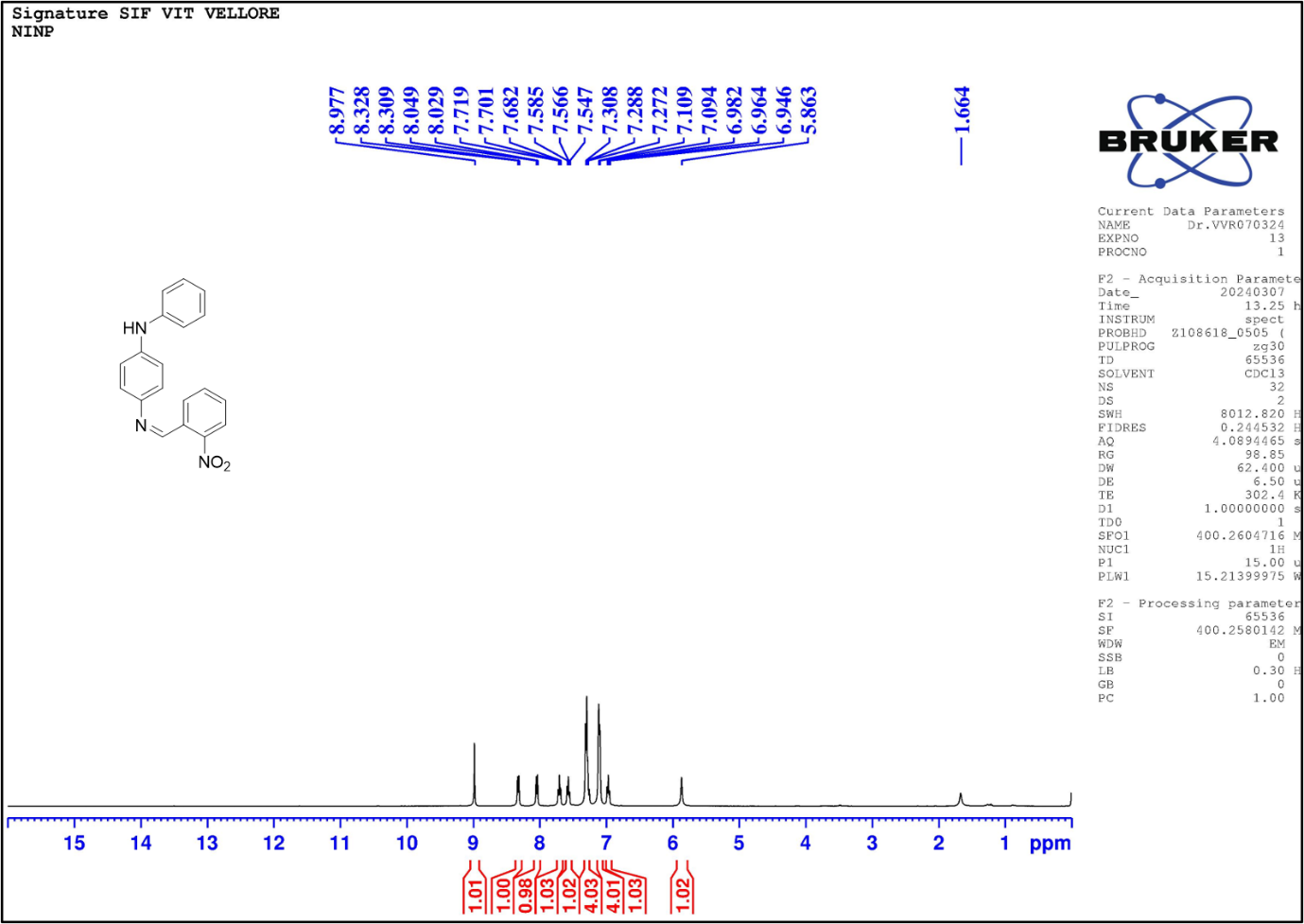
**

**Fig. S21.** ^1^H-NMR spectrum of **L-3** in CDCl_3_ at 25 ^o^C

**
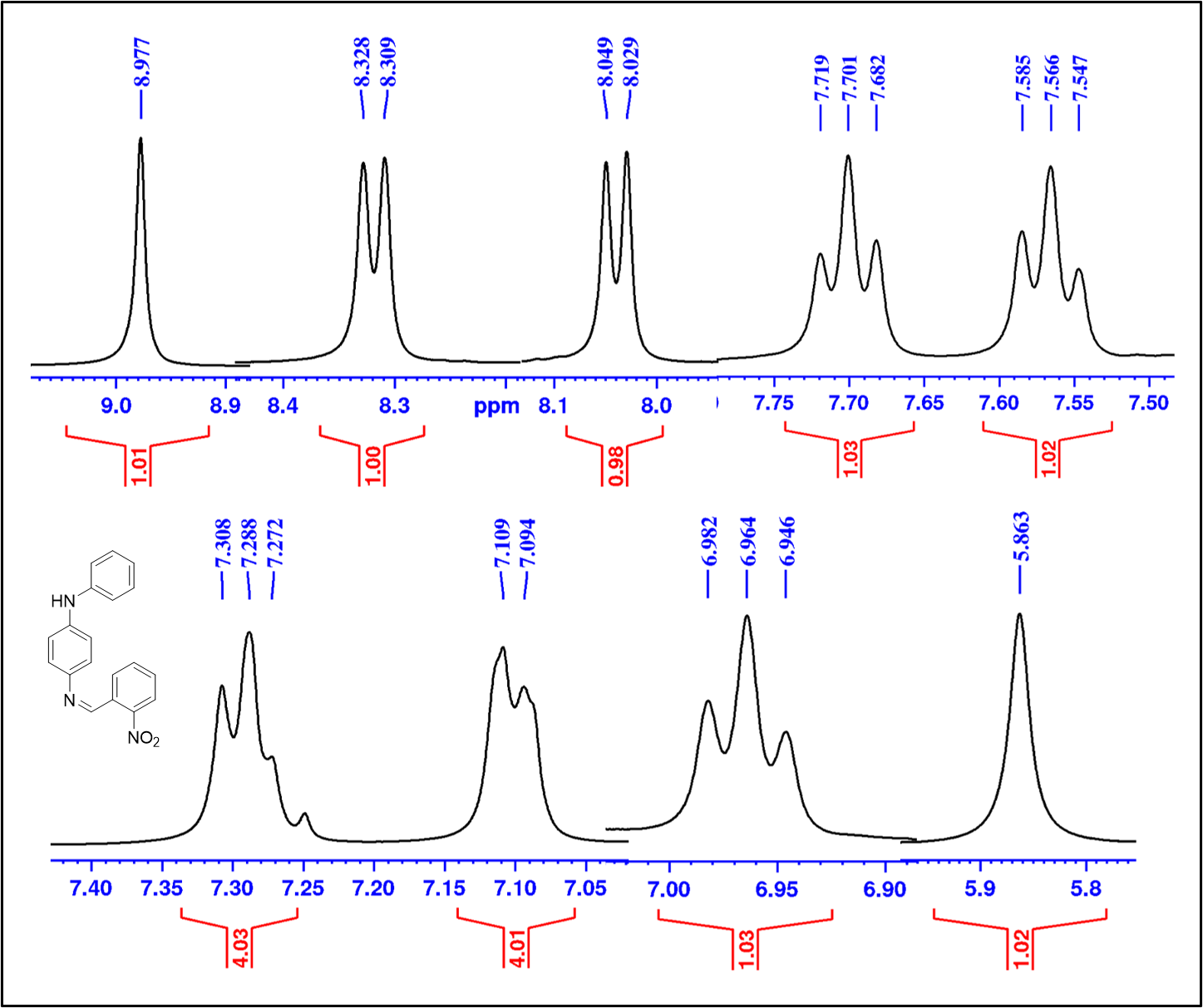
**

**Fig. S22.** ^1^H-NMR enlarged spectrum of **L-3** in CDCl_3_ 25 ^o^C

**
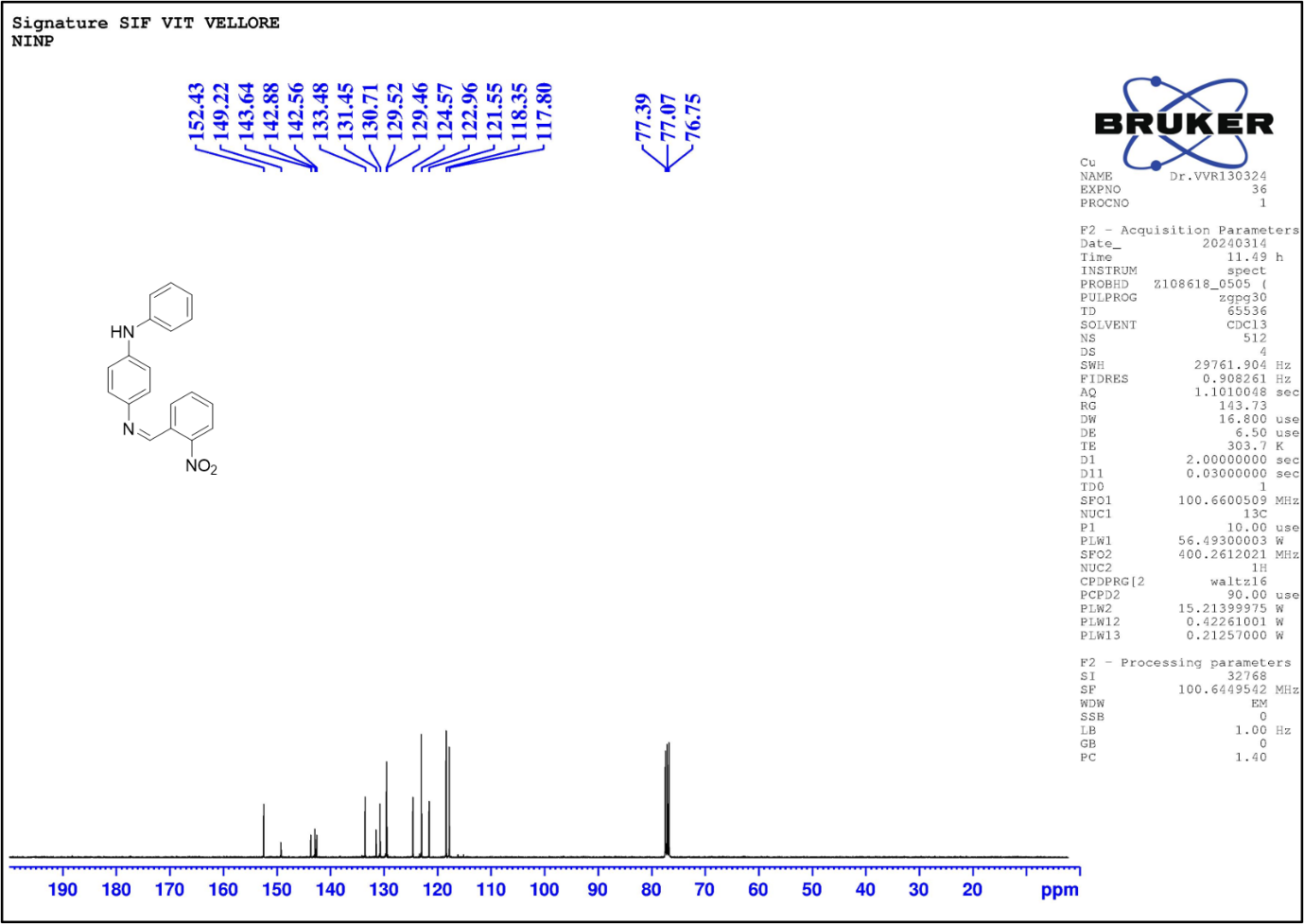
**

**Fig. S23.** ^13^C-NMR spectrum of **L-3** in CDCl_3_ 25 ^o^C


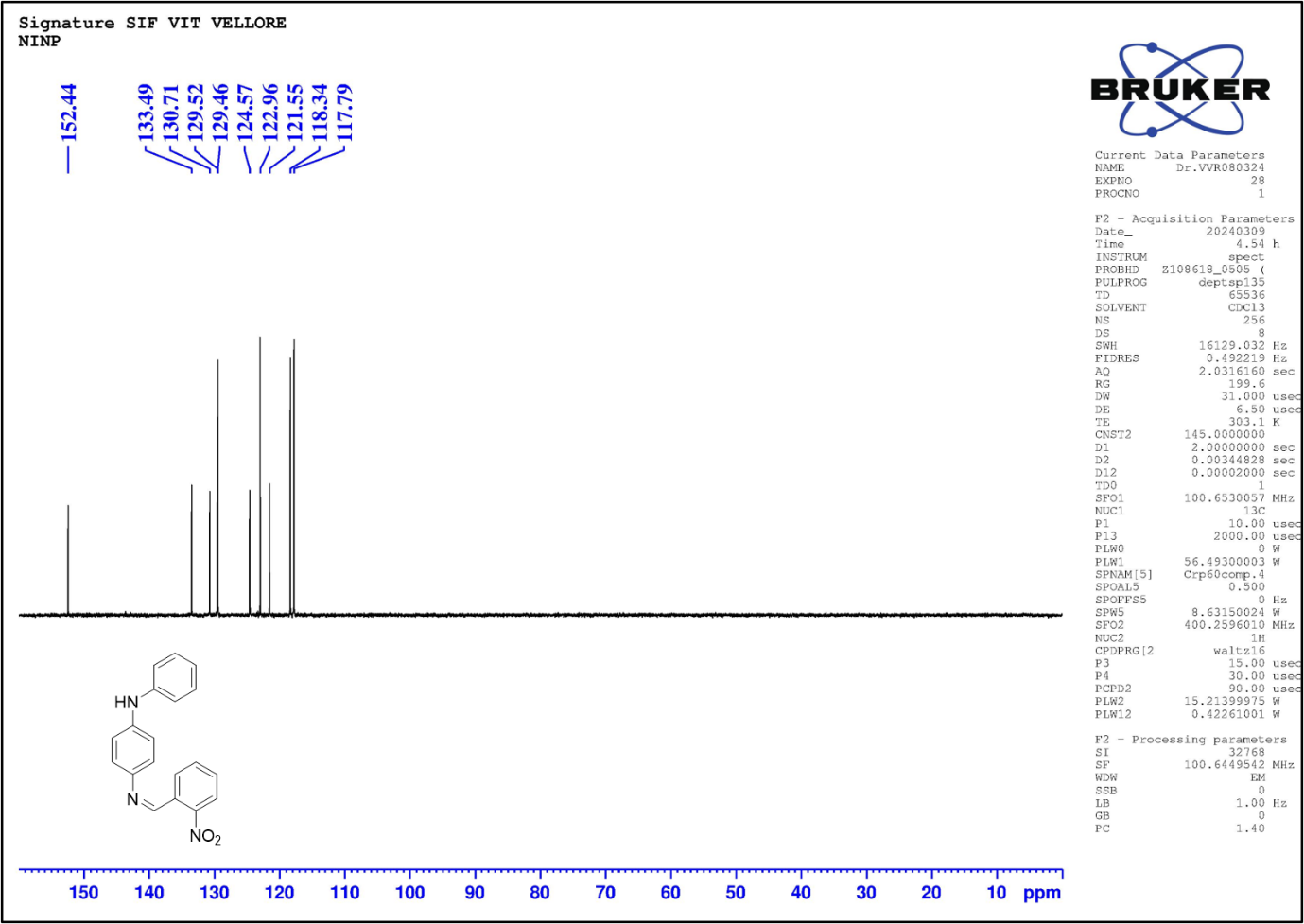


**Fig. S24.** DEPT-135 spectrum of **L-3** in CDCl_3_ 25 ^o^C


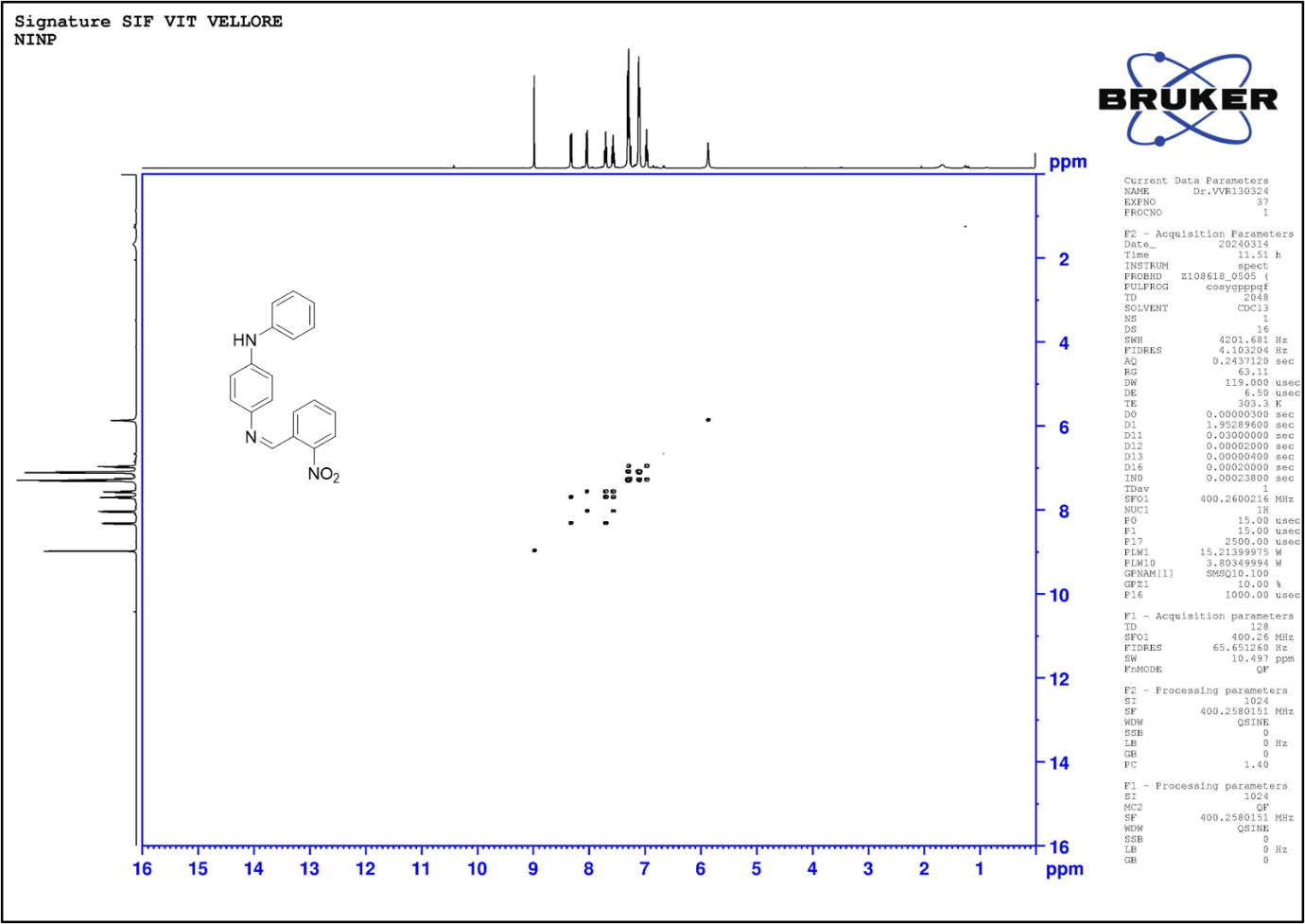


**Fig. S25.** ^1^H-^1^H COSY spectrum of **L-3** in CDCl_3_ at 25 ^o^C


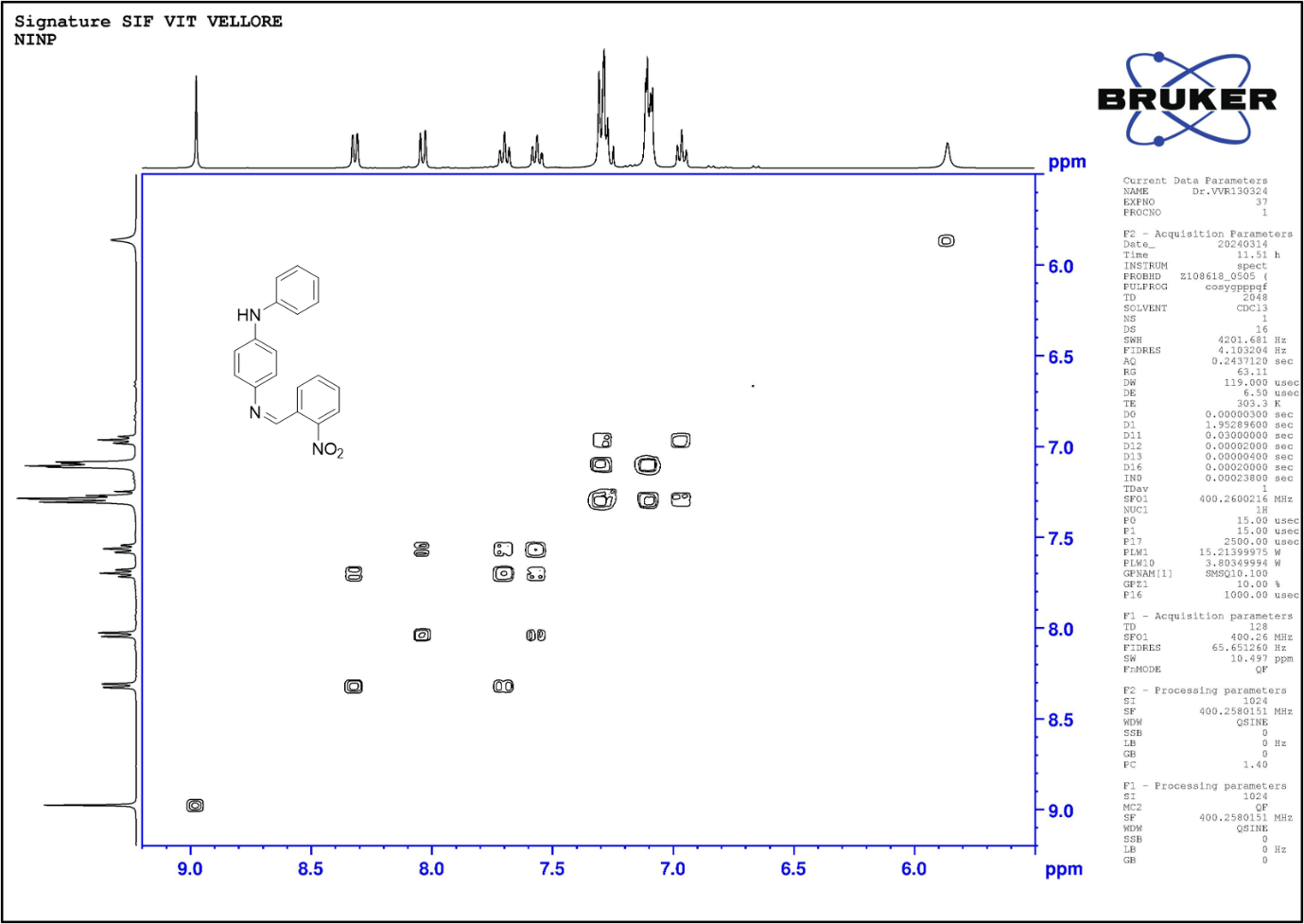


**Fig. S26.** ^1^H-^1^H COSY expanded spectrum of **L-3** in CDCl_3_ at 25 ^o^C

­


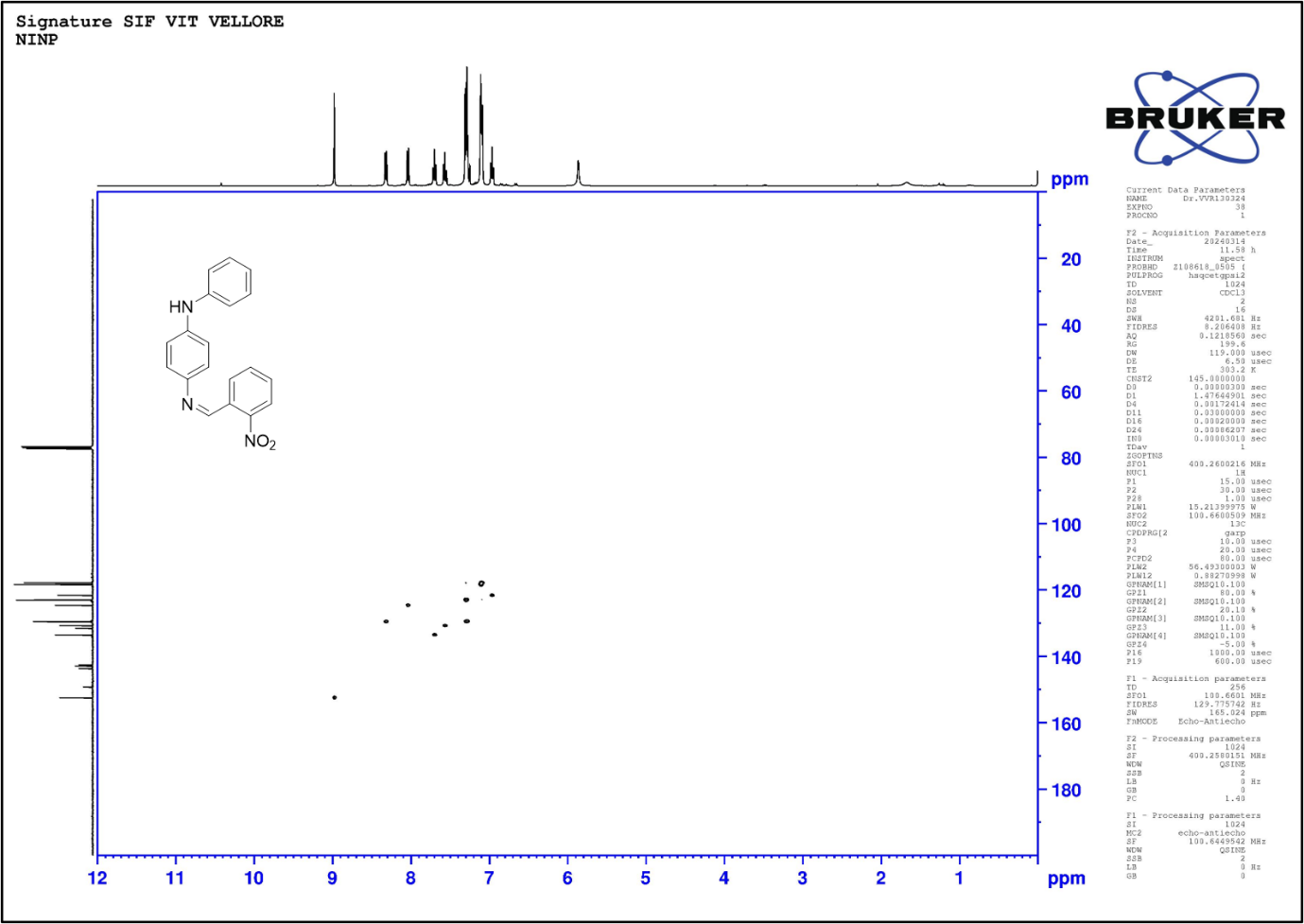


**Fig. S27.**  HSQC spectrum of **L-3** in CDCl_3_ at 25 ^o^C


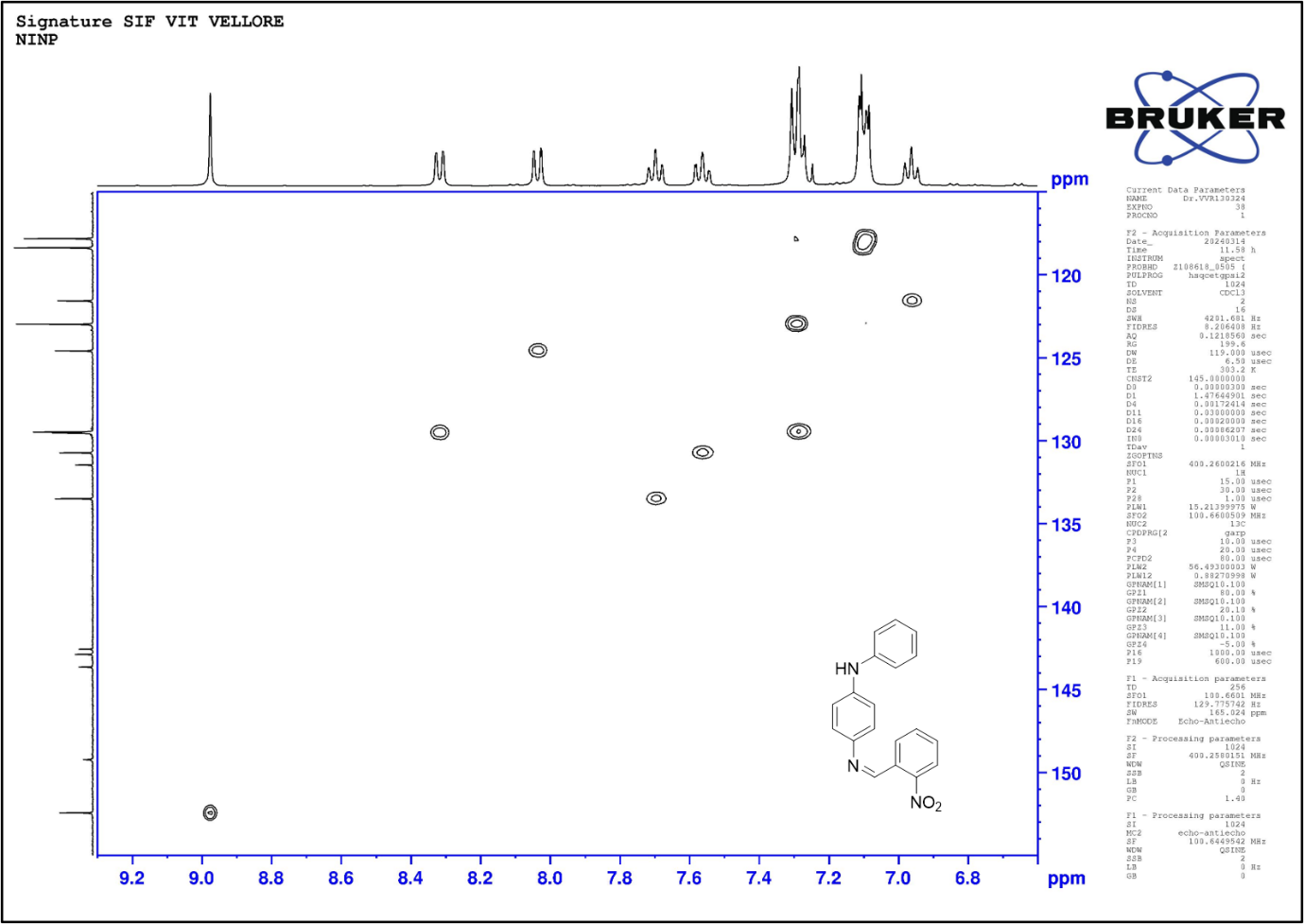


**Fig. S28.**  HSQC expanded spectrum of **L-3** in CDCl_3_ at 25 ^o^C


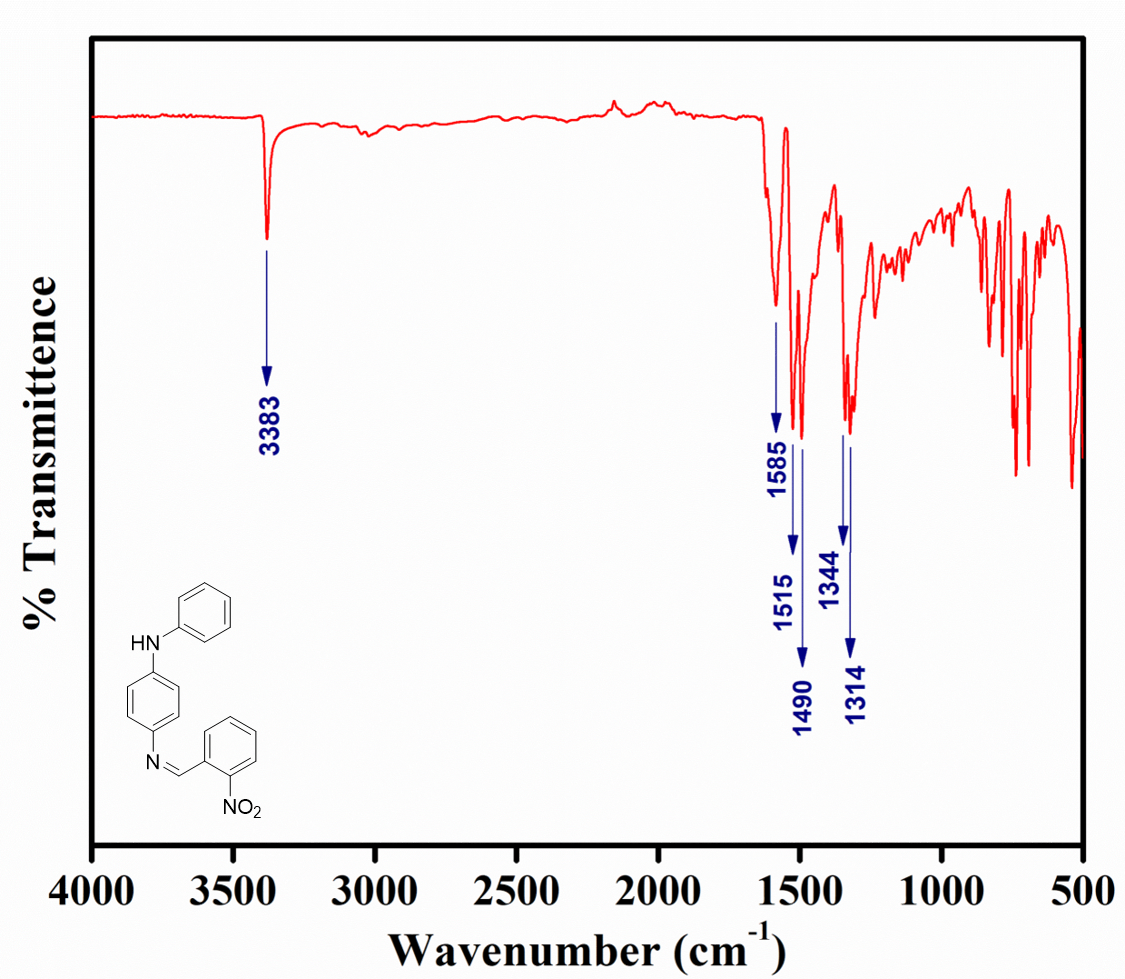


**Fig. S29.** FT-IR spectrum of **L-3**

**
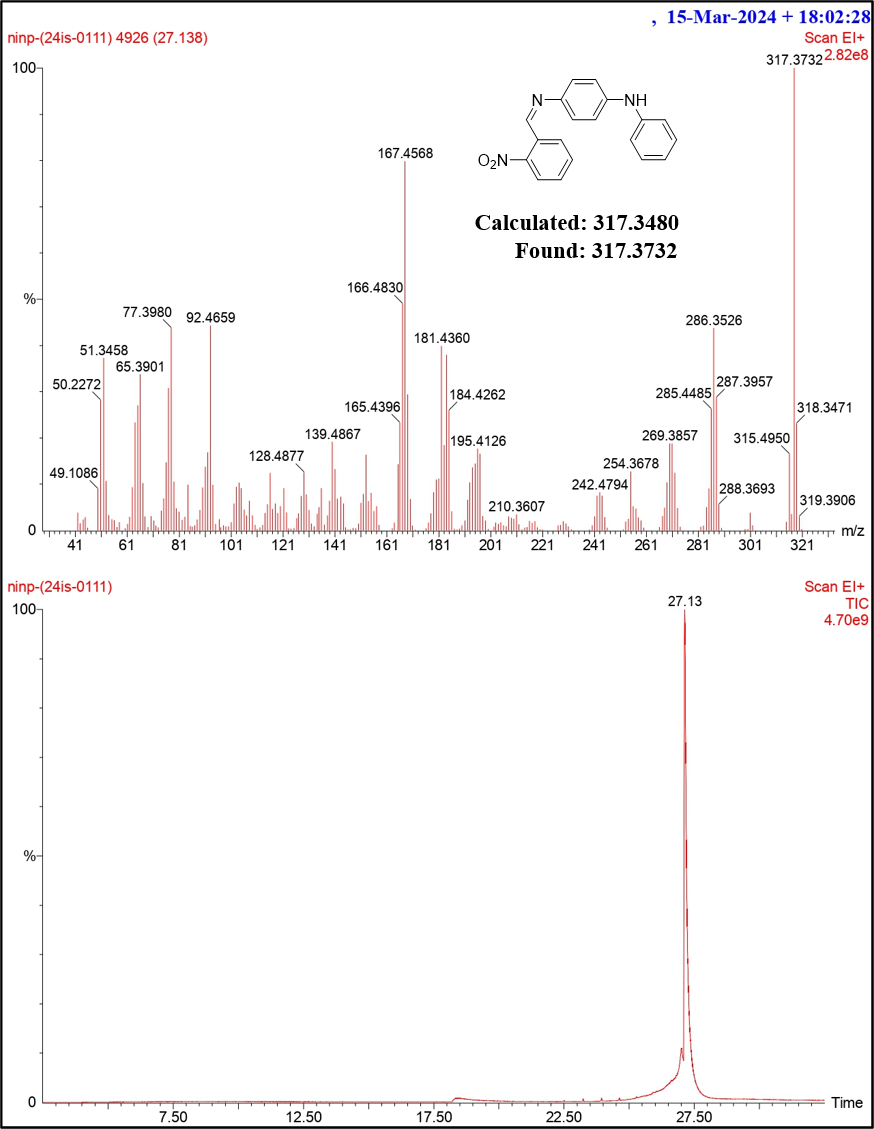
**

**Fig. S30.** GC-Mass spectrum of **L-3**

**
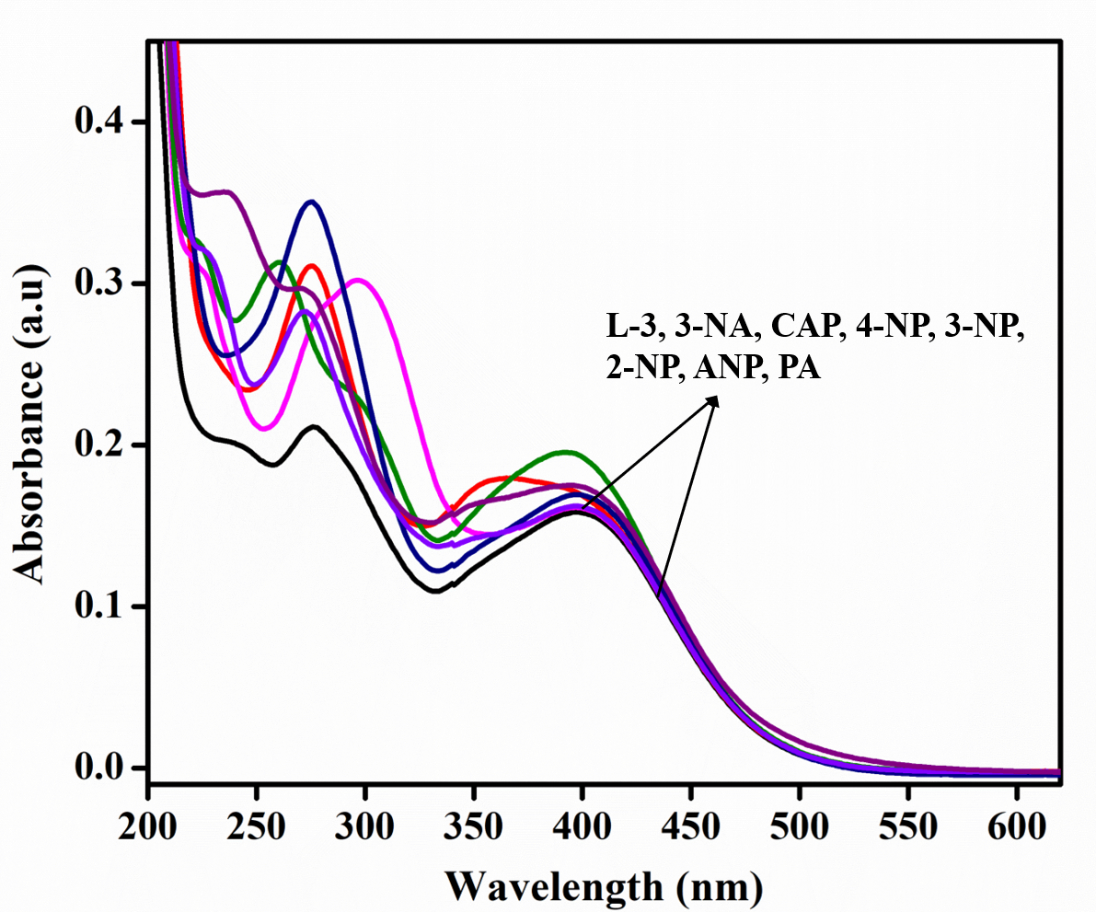
**

**Fig. S31.** Selectivity studies of **L-3** with the other nitro aromatics


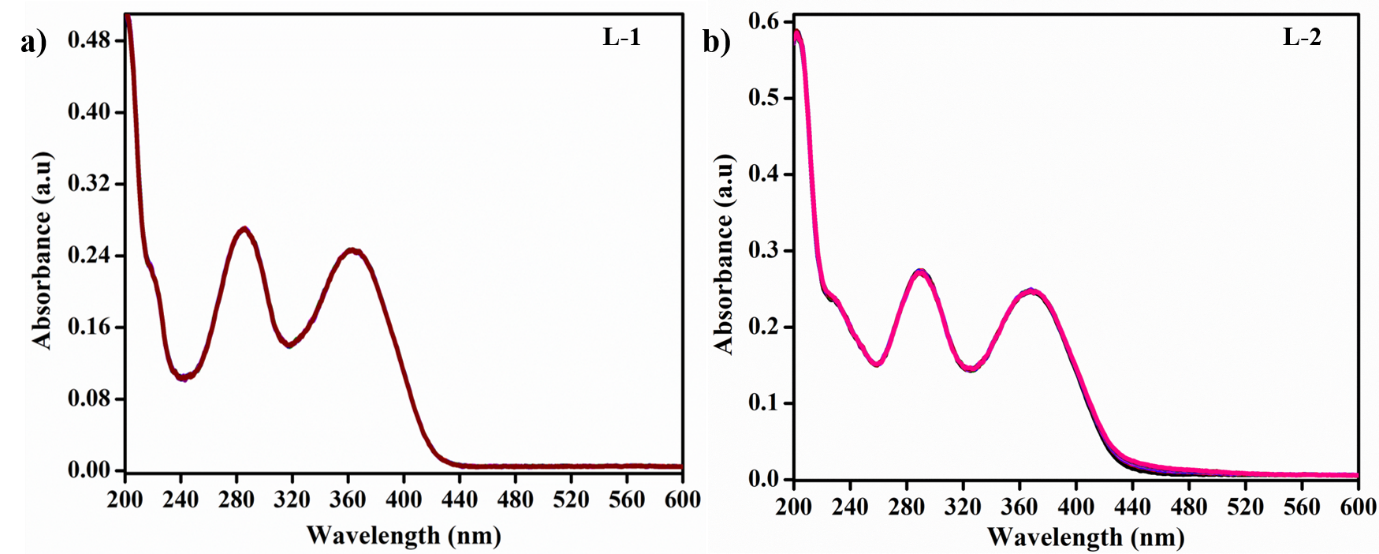


**Fig. S32.** The blank measurement (1x10^-5^) of receptor **L-1** (a) and **L-2** (b)

**Table S1:** Optimized structure of **L-1**, **L-2**, **L-3**, **L-1+PA**, and **L-2+PA** by using basis set B3LYP/6-311+G level of theory

| **Entry** | **Probes** | **Optimized structure** |
| --- | --- | --- |
| 1. |  | 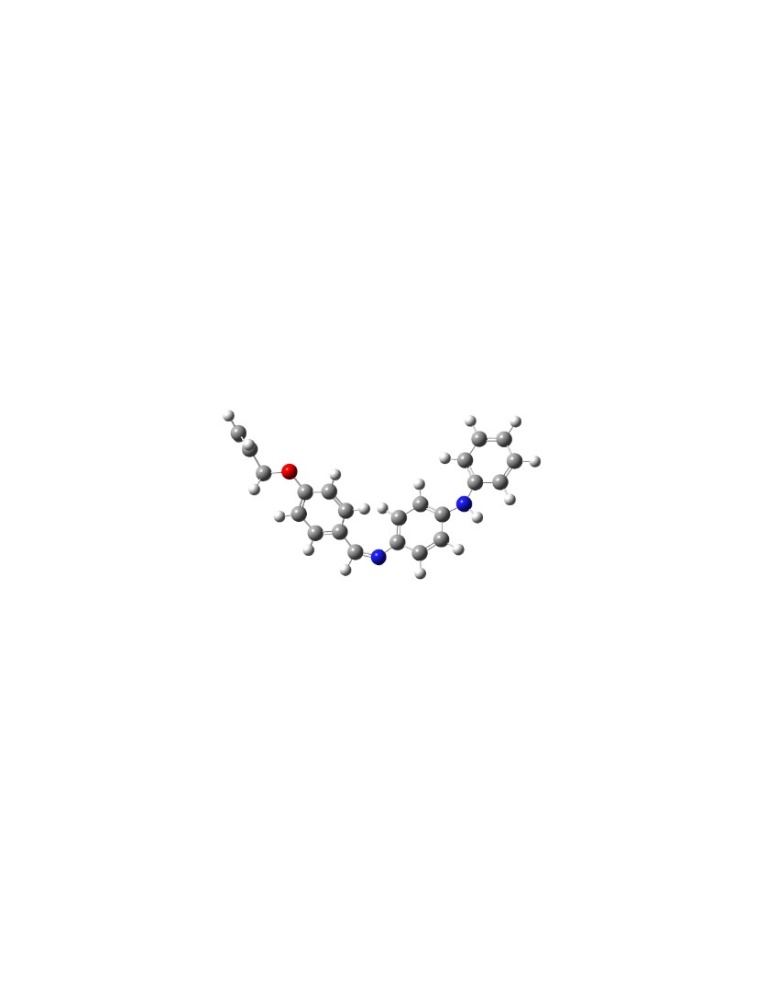 |
| 2. |  | 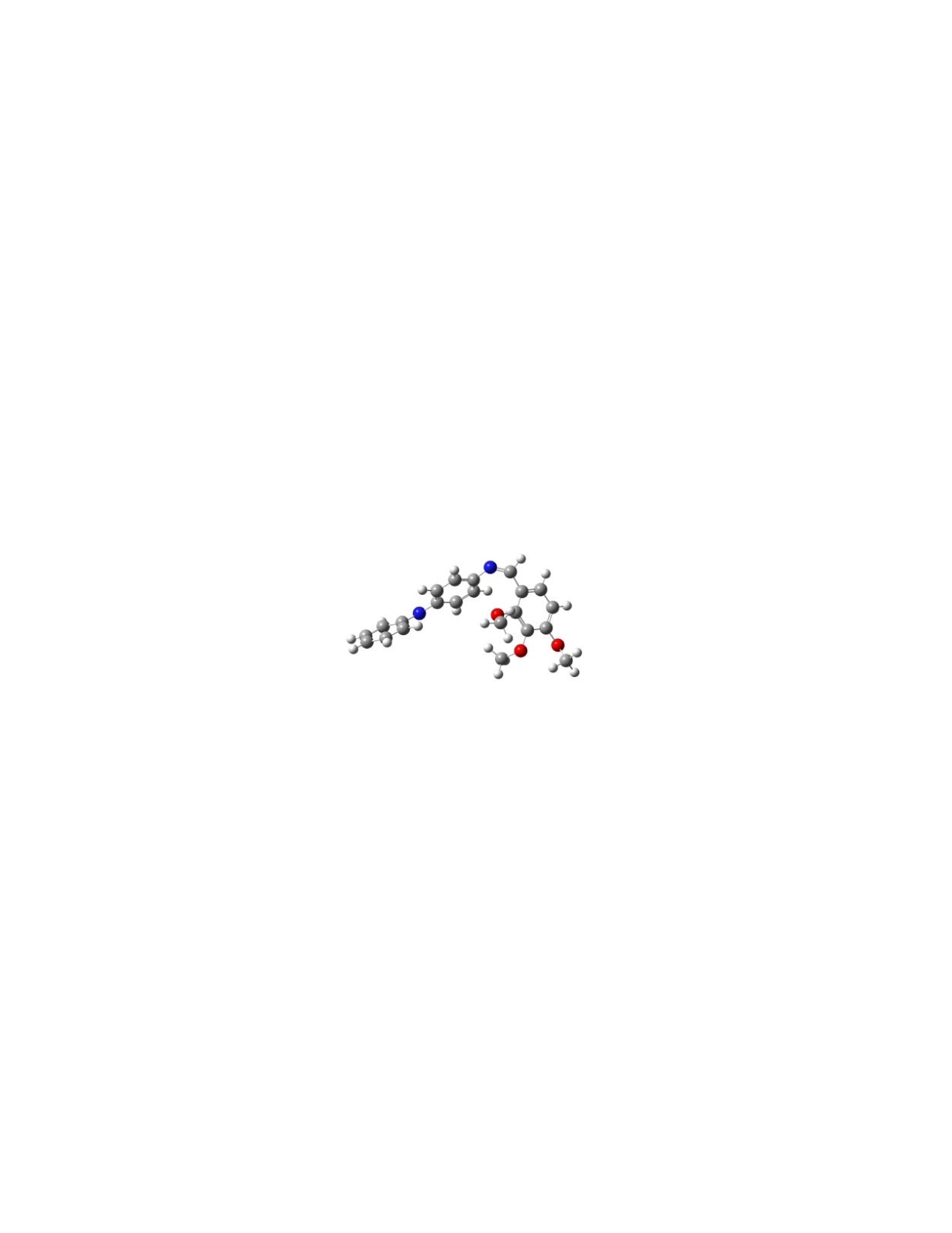 |
| 3. |  | 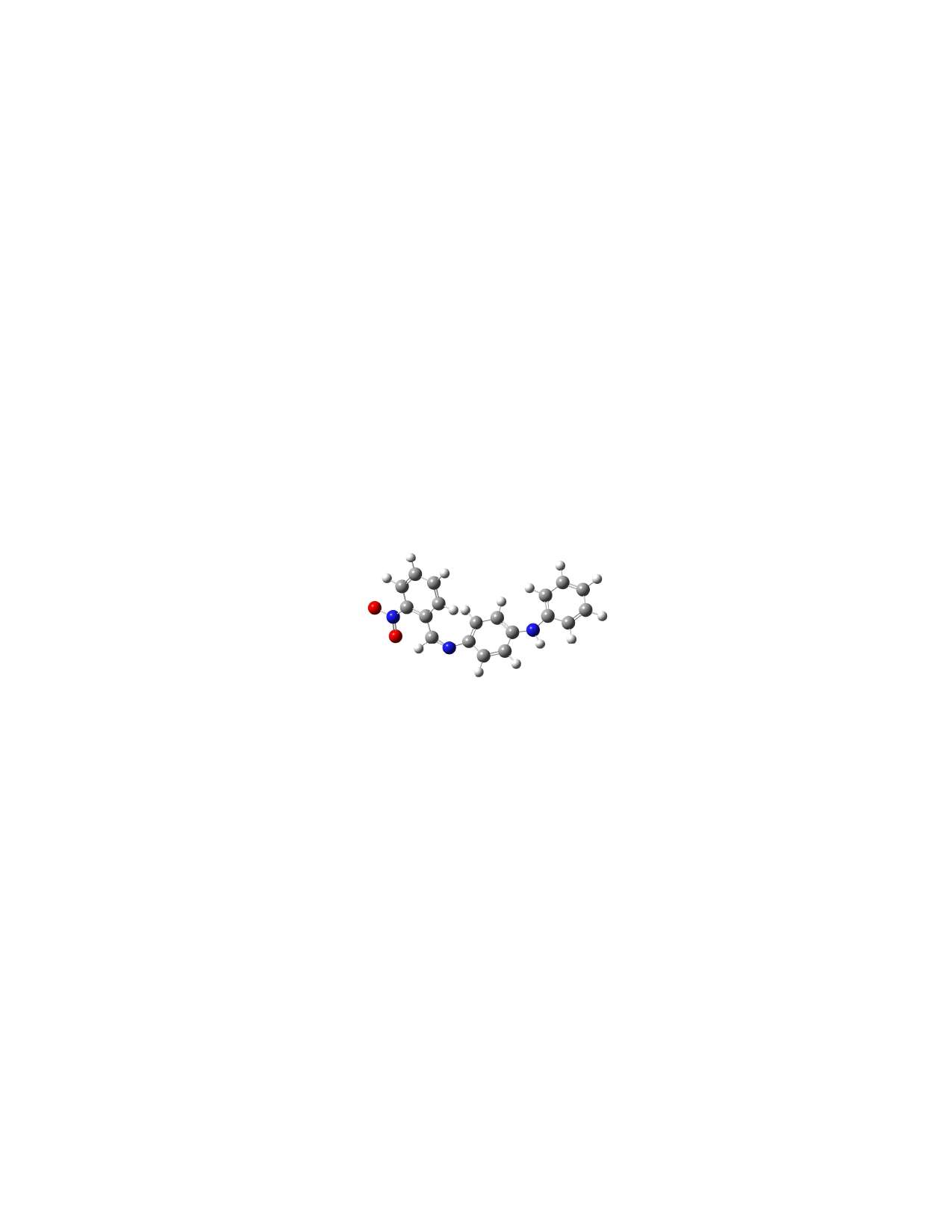 |
| 4. |  | 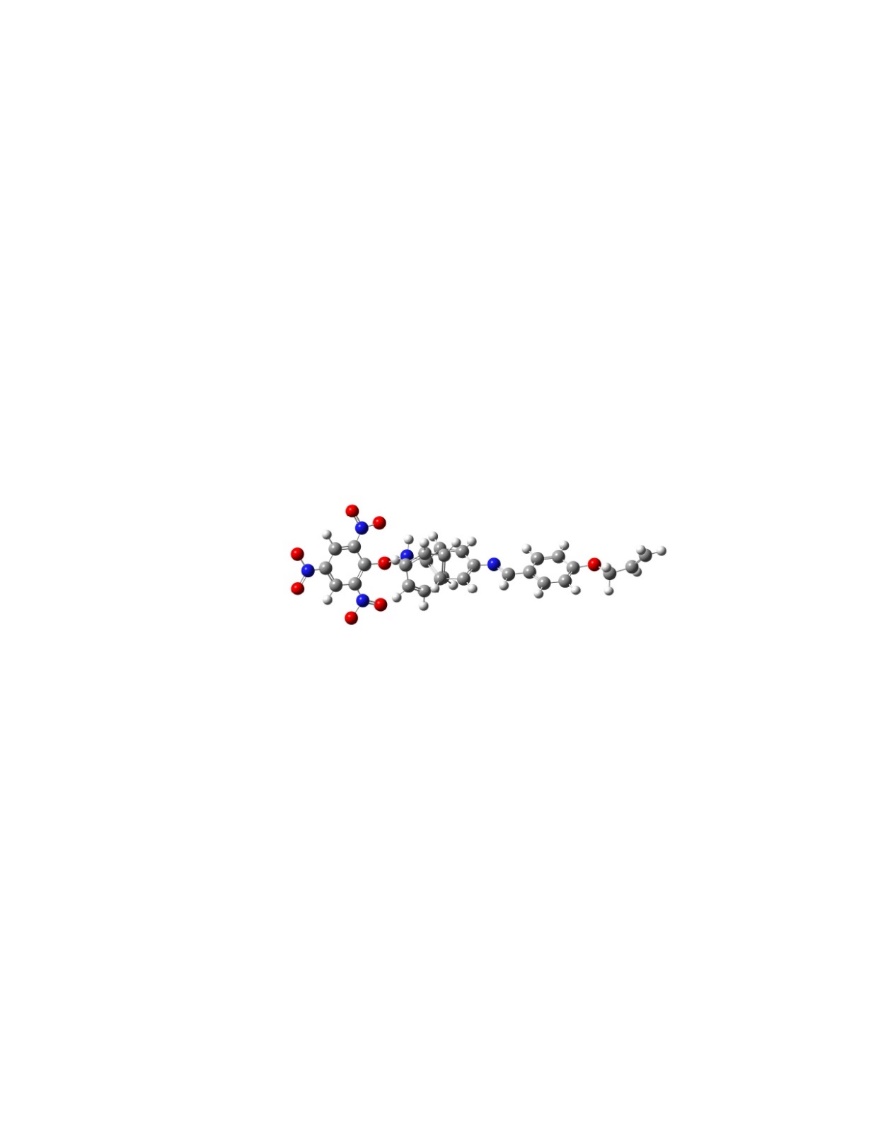 |
| 5. |  | 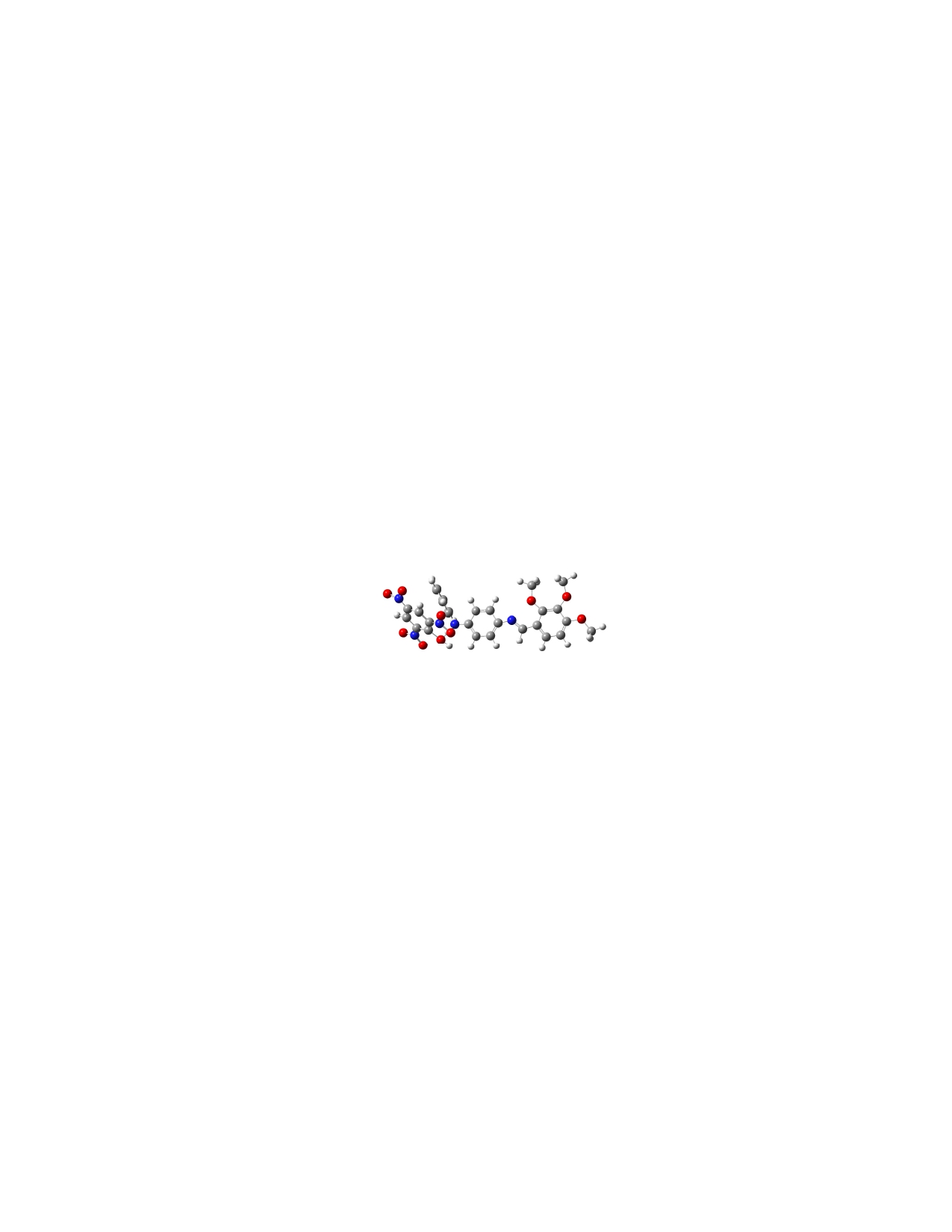 |
